# Supplementary material for: Taxes to red and processed meat to promote sustainable and healthy diets in Mexico
Source: PLoS One. 2025 Jun 27;20(6):e0326616. doi: 10.1371/journal.pone.0326616 (PMC12204545; doi:10.1371/journal.pone.0326616)
Supplement: S1 File — (DOCX) [file pone.0326616.s001.docx]

**Supplementary Information for**

**Taxes to red and processed meat to promote sustainable and healthy diets in Mexico** Kaela Connors^1,2*^, Juan A. Rivera^3^, Peter Alexander^1,2^, Lindsay M. Jaacks^2^, Carolina Batis^4^, Dalia Stern^5^, Martín Lajous^3,6^, M. Arantxa Colchero^7^

^1^ School of Geosciences, University of Edinburgh, Edinburgh, UK

^2^ Global Academy of Agriculture and Food Systems, University of Edinburgh, Midlothian

^3^ Center for Research in Population Health, National Institute of Public Health, Cuernavaca, Morelos, Mexico

^4^ Center for Nutrition and Health Research, National Institute of Public Health, Cuernavaca, Morelos, Mexico

^5^ SECIHTI-Center for Research on Population Health, National Institute of Public Health, Cuernavaca, Morelos, Mexico

^6^ Department of Global Health and Population, Harvard T.H. Chan School of Public Health, Boston, United States

^7^Center for Evaluation and Surveys, National Institute of Public Health, Cuernavaca, Morelos, Mexico

*****Corresponding author:

Kaela Connors

Email: [k.m.connors@sms.ed.ac.uk](mailto:k.m.connors@sms.ed.ac.uk) (KC)

Contents

[**S1 Table – Categorization of Food Expenditures by Food Group in Mexican National Household Income and Expenditure Survey 2018, 2020, and 2022** 3](#_Toc200522940)

[**S2 Table - Categorization of Cuts of Red and Processed Meat according to price in the Mexican National Household Income and Expenditure Survey 2018, 2020, and 2022** 10](#_Toc200522941)

[**S3 Table - Categorization of Red and Processed Meat in the Mexican National Health and Nutrition Survey, 2016** 11](#_Toc200522942)

[**S4 Table- Categorization of Cuts of Meat in the Mexican National Health and Nutrition Survey, 2016** 12](#_Toc200522943)

[**S5 Table - Categorization of Food Groups in the Mexican Teacher’s Cohort, 2006-2014** 13](#_Toc200522944)

[**S6 Table - Sociodemographic characteristics of ENIGH households in Mexico, by survey year and for total sample, 2018-2022** 15](#_Toc200522945)

[**S7 Table– Proportion of Households with Non-Zero Consumption per Food Group in ENIGH by Survey Round and in Total Sample, 2018-2022** 16](#_Toc200522946)

[**S8 Table- Proportion of Households with Non-Zero Consumption per Food Group in ENIGH by Income Quintile, 2018-2022** 17](#_Toc200522947)

[**S9 Table– Average per unit value per Food Group in ENIGH by Survey Round and in Total Sample, 2018-2022** 18](#_Toc200522948)

[**S10 Table– Budget Share of Total Food Expenditure per Food Group in ENIGH by Survey Round and in Total Sample, 2018-2022** 19](#_Toc200522949)

[**S11 Table– Budget Share of Total Food Expenditure per Food Group in ENIGH by Income Quintile, 2018-2022** 20](#_Toc200522950)

[**S12 Table – Own-price elasticity of demand for red meat and other food groups by year in Mexico, 2018-2022** 21](#_Toc200522951)

[**S13 Table- Cross-price elasticity of demand for red meat and other food groups by year in Mexico, 2018-2022** 22](#_Toc200522952)

[**S14 Table - Cross-price elasticity of demand for processed meat and other food groups by year in Mexico, 2018-2022** 23](#_Toc200522953)

[**S15 Table - Own- and cross-price elasticity of demand for red and processed meat with other food groups comparing lowest to highest income quintile in Mexico, 2018-2022** 24](#_Toc200522954)

[**S16 Table – Own- and cross-price elasticity of demand for cuts of meat and other food groups in Mexico, 2018-2022** 25](#_Toc200522955)

[**S2 Figure – Own-price elasticity of demand for expensive and cheap cuts of meat by income quintile in Mexico, 2018-2022** 26](#_Toc200522956)

[**S17 Table- Own- and cross-price elasticity of demand for cuts of meat and other food groups comparing lowest to highest income quintile in Mexico, 2018-2022** 27](#_Toc200522957)

[**S18 Table – Own- and cross-price elasticities of demand for red and processed meat and other food groups in Mexico, 2018-2022** 27](#_Toc200522958)

[**S19 Table– Average daily consumption (g) of red and processed meat in ENSANUT 2016 for adult population (≥18 years), by consumption status, and by income quintile** 28](#_Toc200522959)

[**S20 Table - Correlations between changes in Red, Processed, and Total Meat consumption with Non-Meat Food Groups for Period 1 (2006 -2008) and Period 2 (2008 – 2014) in 1,417 Mexican Women from the Mexican Teachers’ Cohort** 29](#_Toc200522960)

[**S20 Table (cont.) - Correlations between changes in Red, Processed, and Total Meat consumption with Non-Meat Food Groups for Period 1 (2006 -2008) and Period 2 (2008 – 2014) in 1,417 Mexican Women from the Mexican Teachers’ Cohort** 30](#_Toc200522961)

[**S20 Table (cont.) - Correlations between changes in Red, Processed, and Total Meat consumption with Non-Meat Food Groups for Period 1 (2006 -2008) and Period 2 (2008 – 2014) in 1,417 Mexican Women from the Mexican Teachers’ Cohort** 31](#_Toc200522962)

##

## **S1 Table – Categorization of Food Expenditures by Food Group in Mexican National Household Income and Expenditure Survey 2018, 2020, and 2022**

| Food group | Original survey items | English translation |
| --- | --- | --- |
| Dairy | - Leche pasteurizada de vaca - Leche condensada - Leche evaporada - Leche en polvo entera o descremada - Leche modificada o maternizada - Leche no pasteurizada (leche bronca) - Otras leches: de burra, de cabra, de soya - Queso amarillo en rebanadas o para untar - Queso añejo y Cotija - Queso chihuahua - Queso fresco - Queso manchego - Queso Oaxaca o asadero - Otros quesos - Crema - Mantequilla - Bebidas fermentadas de leche - Otros derivados de la leche | • Pasteurized cow's milk  • Condensed milk  • Evaporated milk  • Whole or skimmed milk powder  • Modified or formula milk  • Unpasteurized milk (raw milk)  • Other types of milk: donkey milk, goat milk, soy milk  • Sliced or spreadable yellow cheese  • Aged and cotija cheese  • Chihuahua cheese  • Fresh cheese  • Manchego cheese  • Oaxaca or asadero cheese  • Other types of cheese  • Cream  • Butter  • Fermented milk beverages  • Other milk derivatives |
| Eggs | - Huevo de gallina blanco y rojo - Otros huevos: codorniz, pata, pava etcétera | • White and brown chicken eggs  • Other types of eggs: quail, duck, turkey, etc. |
| Discretionary Foods | - Flanes, gelatinas y pudines en polvo - Cajetas, dulces de leche, jamoncillos y natillas - Helados, nieves y paletas de hielo - Otras golosinas, canasta de dulces - Papas fritas en bolsa o a granel - Pizzas preparadas - Otros alimentos preparados: atole, flautas, guisados, hot dog, sopas, tacos, tamales, tortas, sopes, menudo, pozole, licuados, gelatinas, elotes, etcétera - Pan dulce en piezas (de todo tipo) - Pan dulce empaquetado - Botanas: frituras, palomitas, cheetos, doritos etcétera (excepto papas) - Otros cereales (excepto cereal) - Sopas instantáneas - Pasteles y pastelillos en piezas o a granel, pastel casero - Pasteles y pastelillos empaquetados - Galletas dulces - Galletas saladas - Chocolate en tableta - Chocolate en polvo - Otros chocolates - Concentrados y polvos para preparar bebidas - Refrescos de cola y de sabores - Bebida energética | - Powdered flans, gelatine, and puddings - Cajeta (caramel sauce), milk sweets, *jamoncillos* (traditional candy), and custards - Ice cream, sorbets, and popsicles - Other sweets, assorted candy basket - Bagged or bulk potato chips - Prepared pizzas - Other prepared foods: *atole* (hot corn-based beverage), flautas (deep-fried rolled tortillas), stews, hot dogs, soups, tacos, tamales, sandwiches, sopes (thick tortillas with toppings), menudo (tripe soup), pozole (hominy soup), smoothies, gelatin desserts, corn on the cob, etc. - Pastries (of all kinds) - Packaged pastries - Snacks: fried snacks, popcorn, cheetos, doritos, etc. (excluding potato chips) - Other cereals (excluding cereal) - Instant soups - Cakes and pastries (individual pieces or bulk), homemade cakes - Packaged cakes and pastries - Sweet biscuits - Savoury biscuits - White and brown sugar - Other sugars and honeys - Chocolate bars - Cocoa powder - Other chocolates - Concentrates and powders for beverage preparation - Cola and flavored sodas - Energy drink |
| Grains, roots, and tubers | - Maíz en grano (de todo tipo y color) - Harina de maíz, maicena, fécula, nixtamalizada para tortillas, para atole y harina para tamales - Masa de maíz (de todo tipo y color) - Tortilla de maíz (de todo tipo y color) - Tostadas, raspadas, tostitos, totopos, tlayudas - Otros productos de maíz (excepto cereal) - Harina de trigo - Tortilla de harina - Pasta para sopa - Cereal de maíz, de trigo, de arroz, de avena, de granola, etcétera - Harina para puré de papa - Trigo en grano, otros productos de trigo (excepto cereal) - Arroz en grano - Otros productos de arroz - Pan para sándwich, hamburguesa, hot dog y tostado - Pan blanco: bolillo, telera, baguete, etcétera - Plátano macho y de castilla - Plátano verde y tabasco - Papa - Otros tubérculos | - Corn kernels (of all types and colors) - Cornmeal, cornstarch, nixtamalized corn for tortillas, for *atole*, and flour for tamales - Corn dough (of all types and colors) - Corn tortilla (of all types and colors) - Tostada, scraped corn, tortilla chips, corn chips, tlayudas - Other corn products (excluding cereal) - Wheat flour - Flour tortilla - Soup pasta - Corn cereal, wheat cereal, rice cereal, oat cereal, granola, etc. - Potato puree flour - Wheat grains, other wheat products (excluding cereal) - Rice grains - Other rice products - Sandwich, hamburger, hot dog, and toast bread - White bread: rolls, sandwich bread, baguettes, etc. - Plantains (plantain and castilla varieties) - Green plantains and tabasco plantains - Potato - Other tubers |
| Legumes, nuts, and seeds | - Insectos: chapulines, chinicuiles, escamoles, gusanos de maguey, hormigas (chicatana), jumiles - Frijol en grano - Garbanzo en grano - Haba amarilla o verde en grano - Lenteja en grano - Otras leguminosas en grano - Frijol procesado - Chícharo - Otras leguminosas procesadas - Semillas a granel - Semillas envasadas - Semillas procesadas, soya texturizada | - Insects: grasshoppers (chapulines), maguey worms (chinicuiles), ant eggs (escamoles), maguey worms (gusanos de maguey), ants (chicatana), small shrimp-like creatures (jumiles) - Whole beans - Whole chickpeas - Yellow or green whole fava beans - Whole lentils - Other legumes in grain form - Processed beans - Pea - Other processed legumes - Bulk seeds - Packaged seeds - Processed seeds, textured soy protein |
| Fruits and Vegetables | - Ciruela y jobo - Guayaba - Lima - Limón - Mandarina, nectarina, tangerina - Toronja - Mango - Manzana y perón - Melón - Naranja - Papaya - Pera - Piña - Pitahaya y tuna - Otros plátanos (chiapas, dominico, guineo, manzano, dorado, portalimón y roatan) - Sandía - Uva - Otras frutas: garambullo, granada, higo, jícama, kiwi, etcétera., arreglo frutal - Rábano - Acelgas, espinacas y verdolagas, quintolines, quelites y pipica - Aguacate - Ajo - Brócoli - Calabacita y calabaza - Cebolla - Chayote - Chile jalapeño - Chile poblano - Chile serrano - Otros chiles - Cilantro - Col y repollo - Ejote | - Plum and jocote - Guava - Lime - Lemon - Mandarin, nectarine, tangerine - Grapefruit - Mango - Apple and pear - Cantaloupe - Orange - Papaya - Pear - Pineapple - Dragon fruit and prickly pear - Other bananas (chiapas, dominico, guineo, manzano, dorado, portalimón, and roatan) - Watermelon - Grape - Other fruits : garambullo, pomegranate, fig, jicama, kiwi, etc., fruit arrangement - Radish - Swiss chard, spinach, purslane, quelites, and pipica - Avocado - Garlic - Broccoli - Zucchini and pumpkin - Onion - Chayote - Jalapeño pepper - Poblano pepper - Serrano pepper - Other peppers - Cilantro - Cabbage and lettuce - Green bean |
| Fruits and Vegetables | - Elote - Epazote - Jitomate - Lechuga - Nopal - Pepino - Perejil y yerbabuena - Tomate verde - Zanahoria - Betabel y camote - Otras verduras, pápalo, aceitunas, flor de calabaza, chaya - Germinados de maíz, de soya, de trigo, de alfalfa - Chiles envasados - Chile secos o en polvo - Verduras y legumbres envasadas, aceitunas, verduras encurtidas - Verduras y legumbres congeladas - Frutas: anona, chirimoya, guanábana, cereza, frambuesa, fresa, zarzamora, chabacano, durazno, melocotón, chicozapote, mamey, ciruela, jobo, guayaba, lima, limón, mandarina, nectarina, tangerina, toronja, mango, manzana, perón, melón, naranja, papaya, pera, piña, pitahaya, tuna, plátano macho y de castilla, plátano verde y tabasco, otros plátanos (chiapas, dominico, guineo, manzano, dorado, portalimón y roatan), sandía, uva, otras frutas (garambullo, granada, higo, jícama, kiwi, etcétera), arreglo frutal - Verduras y legumbres envasadas, aceitunas, verduras encurtidas - Frutas en almíbar y conserva - Frutas cristalizadas, enchiladas y secas - Hongos frescos: champiñones, huitlacoche y setas - Anona, chirimoya, guanábana - Cereza, frambuesa, fresa, zarzamora - Chabacano, durazno, melocotón - Chicozapote y mamey | - Corn on the cob - Epazote - Tomato - Lettuce - Nopal (prickly pear cactus) - Cucumber - Parsley and mint - Green tomato - Carrot - Beetroot and sweet potato - Other vegetables: pápalo (a type of herb), olives, squash blossoms, chaya (tree spinach) - Corn, soy, wheat, and alfalfa sprouts - Canned peppers - Dried or powdered chili peppers - Canned vegetables and legumes, olives, pickled vegetables - Frozen vegetables and legumes - Fruits: custard apple, cherimoya, soursop, cherry, raspberry, strawberry, blackberry, loquat, peach, sapote, mamey, plum, jocote, guava, lime, lemon, mandarin, nectarine, tangerine, grapefruit, mango, apple, pear, pineapple, dragon fruit, prickly pear, plantains (plantain and castilla varieties), green plantains, tabasco plantains, other plantains (chiapas, dominico, guineo, manzano, dorado, portalimón, and roatan), watermelon, grape, other fruits (garambullo, pomegranate, fig, jicama, kiwi, etc.), fruit arrangement - Canned vegetables and legumes, olives, pickled vegetables - Fruits in syrup and preserves - Crystallized, spiced, and dried fruits - Fresh mushrooms: mushrooms, huitlacoche (corn smut), and wild mushrooms - Custard apple, cherimoya, soursop - Cherry, raspberry, strawberry, blackberry - Loquat, peach, sapote - Chicozapote and mamey |
| Red Meat | - Bistec de res (de cualquier parte que se saque) - Arrachera, filete - Milanesa de res - Chamorro de res - Chuleta de costilla de res - Agujas, aldilla, chambarete, diezmillo, espinazo, fajilla de res para asar, retazo, tampiqueña - Cocido de res - Cortes especiales de res - Hamburguesas de res para asar - Molida de res - Pulpa de res en trozo - Carne de otras partes de la res - Bistec de puerco (de cualquier parte que se saque) - Pierna de puerco en trozo - Pulpa de puerco en trozo - Molida de puerco - Costilla y chuleta de puerco - Espaldilla de puerco - Codillo de puerco - Carne de otras partes del puerco - Carne enchilada - Borrego y carnero - Chivo y cabrito - Otras carnes: caballo, conejo, iguana, jabalí, rana, tortuga, venado - Carnitas - Barbacoa y birria - Vísceras de res - Vísceras de puerco | - Beef steak (from any part) - Skirt steak, fillet - Breaded beef - Beef shank - Rib chop of beef - Beef chuck, brisket, shin, sirloin tip, backbone, beef flank for grilling, trimmings, tampiqueña (a style of beef dish) - Boiled beef - Special beef cuts - Beef patties for grilling - Ground beef - Chunk of beef pulp - Meat from other parts of the beef - Pork steak (from any part) - Pork leg in pieces - Pork pulp in pieces - Ground pork - Pork rib and chop - Pork shoulder - Pork knuckle - Meat from other parts of the pork - Seasoned meat - Lamb and mutton - Goat and little goat - Other meats: horse, rabbit, iguana, wild boar, frog, turtle, deer - Carnitas (fried pork) - Barbecue and birria (a Mexican stew) - Beef organs - Pork organs |
| Processed Meat | - Chicharrón de puerco - Chorizo con cualquier condimento y color y longaniza - Chuleta ahumada de puerco - Machaca y carne seca - Mortadela, queso de puerco y salami, bolonia de carnes surtidas - Lardo procesado (tocino) - Salchichas y salchichón - Otras carnes procesadas, cueritos - Chorizo de pollo, jamón y nugget, salchicha, mortadela, etcétera - Jamón de puerco | - Pork cracklings - Sausage with any seasoning, color, and longaniza - Smoked pork chop - Dried shredded meat and beef jerky - Mortadella, pork cheese, and salami, assorted meat bologna - Processed lard (bacon) - Sausages and salchichón (a type of sausage) - Other processed meats, pork rinds - Chicken sausage, ham, and nuggets, hot dog, mortadella, etc. - Ham |
| Seafood | - Pescado entero limpio y sin limpiar - Filete de pescado - Atún enlatado - Salmón y bacalao procesado - Pescado ahumado, seco, nugget, sardina, etcétera - Anguilas, angulas, hueva de pescado, mantarraya, pejelagarto, etcétera - Camarón fresco - Mariscos frescos - Mariscos procesados | - Whole fish, cleaned and uncleaned - Fish fillet - Canned tuna - Processed salmon and cod - Smoked fish, dried fish, fish nuggets, sardines, etc. - Eels, elvers, fish roe, stingray, garfish, etc. - Fresh shrimp - Fresh seafood - Processed seafood |
| Poultry | - Pierna, muslo o pechuga de pollo con hueso - Pierna, muslo o pechuga de pollo sin hueso - Pollo entero o en piezas (excepto, pierna, muslo y pechuga) - Otras aves - Pollo rostizado - Vísceras y otras partes del pollo | - Chicken leg, thigh, or breast with bone - Chicken leg, thigh, or breast without bone - Whole chicken or in pieces (excluding leg, thigh, and breast) - Other poultry - Roasted chicken - Innards and other parts of the chicken |
| Other Foods | - Azúcar blanca y Morena - Otras azúcares y mieles - Miel de abeja - Café tostado en grano molido - Café tostado soluble, capuchino - Flor y hojas para té - Té soluble (cualquier sabor) - Canela - Clavo - Yerbas de olor - Concentrados de pollo, tomate, camarón y res - Mostaza - Pimienta - Sal - Salsas dulces y picantes - Vinagre - Otros aderezos, especies y salsas, vainilla - Agua natural embotellada - Agua mineral, quina, desmineralizada con o sin sabor - Aceite de coco, oliva, soya - Otros aceites: de bacalao, de tiburón, de tortuga, enjundia - Mayonesa - Mole en pasta o en polvo - Agua preparada y jugos naturales - Jugos y néctares envasados - Ates, crema de cacahuate, jaleas, mermelada - Aceite vegetal: canola, cártamo, girasol, maíz, etcétera - Margarina - Manteca de puerco - Manteca vegetal - Hielo, Jarabe natural, lechugilla, sangrita, tascalate | - Honey - Ground roasted coffee beans - Instant roasted coffee, cappuccino - Tea leaves and tea bags - Instant tea (any flavor) - Cinnamon - Cloves - Aromatic herbs - Chicken, tomato, shrimp, and beef concentrates - Mustard - Pepper - Salt - Sweet and spicy sauces - Vinegar - Other dressings, spices, and sauces, vanilla - Bottled natural water - Mineral water, tonic water, demineralized water with or without flavor - Coconut oil, olive oil, soybean oil - Other oils: cod liver oil, shark liver oil, turtle oil, suet - Mayonnaise - Mole paste or powder - Prepared water and natural juices - Packaged juices and nectars - Fruit pastes, peanut butter, jellies, jams - Vegetable oil: canola, safflower, sunflower, corn, etc. - Margarine - Pork lard - Vegetable shortening - Ice, natural syrup, lechugilla (a traditional Mexican beverage), sangrita (a spicy drink), tascalate (a traditional Mexican beverage) |
| Excluded | - Cigarros - Puros - Tabaco en hoja y picado - Alimento para animales domésticos - Alimento para animales para uso del hogar - Alimentos y/o bebidas en paquete, despensa de alimentos que otorgan otros hogares - Despensa de alimentos que otorgan organizaciones privadas o de gobierno - Otros gastos relacionados con la preparación de alimentos, cal para nixtamal - Molienda de nixtamal - Cereal de arroz, avena, plátano, manzana, mixto para bebé - Papillas para bebé - Jugos de frutas y verduras de cualquier combinación para bebé | - Cigarettes - Cigars - Loose and shredded tobacco leaves - Pet food. - Animal feed for household use. - Packaged foods and/or beverages, pantry items provided by other households - Pantry of foods provided by private or government organizations. - Other expenses related to food preparation, lime for nixtamalization - Grinder for corn masa - Rice cereal, oatmeal, banana, apple, baby mix - Baby purees - Fruit and vegetable juices of any combination for babies |
|  | - Cerveza - Vino de mesa blanco, rosado, tinto - Otras bebidas alcohólicas: champaña - Whisky Aguardiente, alcohol de caña, charanda, mezcal - Agua ardiente, alcohol de caña, charanda, mezcal - Cognac y brandy - Anís (licor) - Jerez - Licor de frutas o cremas - Néctar de agave, pulque, tlachique, tepache, tuba y bebidas fermentadas de maíz - Ron añejo, ron blanco con limón - Rompope - Sidra blanca y rosada - Tequila añejo, azul y blanco - Vodka - Desayuno - Comida - Cena - Entrecomidas - Otros eventos fuera de casa | - Beer - White, rosé, red table wine - Other alcoholic beverages: champagne - Whiskey - Aguardiente, cane alcohol, charanda, mezcal - Cognac and brandy - Anise (liquor) - Sherry - Fruit liqueur or creams - Agave nectar, pulque, tlachique, tepache, tuba, and fermented corn drinks - Aged rum, white rum with lemon - Eggnog - White and pink cider - Aged, blue, and white tequila - Vodka - Breakfast - Lunch - Dinner - Snacks - Other events outside the home |

**S1 Figure– Price increase ratios (%) for red and processed meat to achieve Mexican Dietary Guidelines 2023 and EAT-Lancet Dietary Targets for Mexican Adult ( ≥ 18 years) Population**


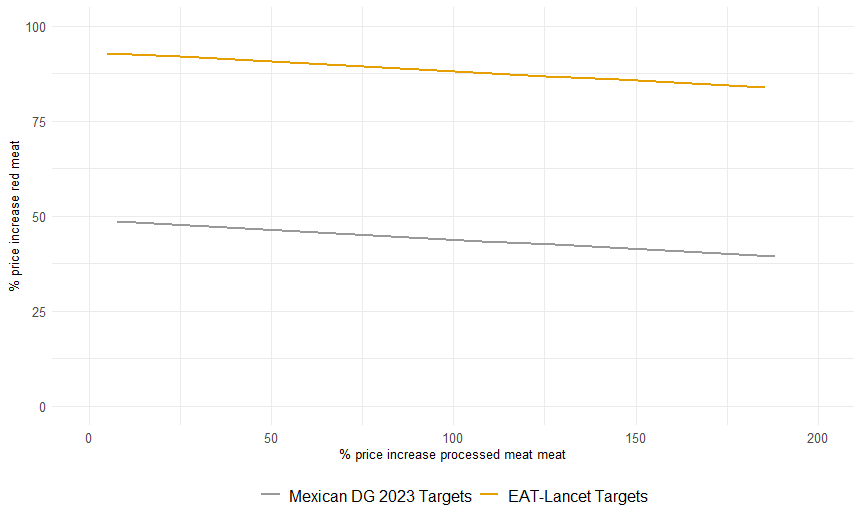


Based on own-and cross-price elasticities for red and processed meat assuming linearity in relationship between price and demand for red and processed meat.

Price increase ratios represent those that would be required to achieve Mexican DG 2023 Targets for red and processed meat (30g/day and 0g/day, respectively) and to achieve EAT-Lancet Dietary Targets for red and processed meat (14g/day and 0g/day, respectively).

A percent reduction of 34% and 100% for red and processed meat was calculated for the Mexican adult population based on current consumption levels to achieve Mexican DG 2023 targets.

A percent reduction of 69.2% and 100% for red and processed meat was calculated for the Mexican adult population based on current consumption levels to achieve EAT-Lancet Dietary Targets.

# **S2 Table - Categorization of Cuts of Red and Processed Meat according to price in the Mexican National Household Income and Expenditure Survey 2018, 2020, and 2022**

| Categorization of Cut of Meat based on Price per Unit | Original survey items | English translation |
| --- | --- | --- |
| Cheaper Cut of Meat | - Bistec de puerco (de cualquier parte que se saque) - Carne de otras partes del puerco Chamorro de res - Chorizo con cualquier condimento y color y longaniza - Chorizo de pollo, jamón y nugget, salchicha, mortadela, etcétera Chuleta ahumada de puerco Cocido de res Codillo de puerco Costilla y chuleta de puerco Espaldilla de puerco - Jamón de puerco - Molida de puerco - Mortadela, queso de puerco y salami, bolonia de carnes surtidas Pierna de puerco en trozo - Pulpa de puerco en trozo Salchichas y salchichón - Vísceras de puerco - Vísceras de res | - Pork steak (from any part) - Meat from other parts of the pig - Beef shank - Sausage with any seasoning, color, and longaniza - Chicken sausage, ham and nugget, sausage, bologna, etc. - Smoked pork chop - Beef stew - Pork knuckle - Pork ribs and chop - Pork shoulder - Pork ham - Ground pork - Mortadella, pork cheese, and salami, assorted meat bologna - Pork leg in pieces - Pork pulp in pieces - Sausages and salami - Pork innards - Beef innards |
| Expensive Cut of Meat | - Agujas, aldilla, chambarete, diezmillo, espinazo, fajilla de res para asar, retazo, tampiqueña - Arrachera, filete - Barbacoa y birria - Bistec de res (de cualquier parte que se saque) - Borrego y carnero - Carne de otras partes de la res - Carne enchilada - Carnitas - Chicharrón de puerco - Chivo y cabrito - Chuleta de costilla de res - Cortes especiales de res - Hamburguesas de res para asar - Lardo procesado (tocino) - Machaca y carne seca - Milanesa de res - Molida de res - Otras carnes procesadas, cueritos - Otras carnes: caballo, conejo, iguana, jabalí, rana, tortuga, venado - Pulpa de res en trozo | - Needles, blade, shin, chuck, backbone, beef fajita for grilling, trimmings, tampiqueña - Skirt steak, fillet - Barbacoa and birria - Beef steak (from any part) - Lamb and mutton - Meat from other parts of the beef - Spiced meat - Carnitas - Pork cracklings - Goat and kid - Beef rib chop - Special beef cuts - Grill-ready beef burgers - Processed lard (bacon) - Machaca and dried meat - Beef Milanese - Ground beef - Other processed meats, pork rinds - Other meats: horse, rabbit, iguana, wild boar, frog, turtle, venison - Beef pulp in pieces |
| Items include both red and processed meat. Cheaper cuts of meat were defined as individual items with a price per unit ≤ 114.00 MXN (the weighted national average price per unit across all three survey rounds for red and processed meat combined). Expensive cuts of meat were defined as individual items with a price per unit > 114.00- MXN. | | |

# **S3 Table - Categorization of Red and Processed Meat in the Mexican National Health and Nutrition Survey, 2016**

| Food group | Original survey items | English translation |
| --- | --- | --- |
| Unprocessed Red Meat | - Res, aguayon - Res, agujas - Carnero, magra con hueso - Carnero, magra sin hueso - Cerdo, carne magra (pierna o lomo sin grasa) - Cerdo, carne promedio - Cerdo, semigrasosa - Res, carne molida especial - Res, carne molida regular - Res, carne grasosa con hueso - Res, carne grasosa sin hueso - Res, carne magra (sin grasa ni hueso) - Res, carne semigrasosa (tipo bistec) - Carnero, carne grasosa - Res, chambarete - Res, Corazón - Cerdo, costilla semigrasosa - Cerdo, espaldilla - Res, falda - Res, filete - Res, hígado - Res, estomago e intestinos crudos - Res, patas - Cerdo, patas frescas - Res, pescuezo - Res, sesos - Res, tripas - Venado, carne magra cruda - Cerdo, carne molida - Carne de res cocida - Carne de cerdo cocida | - Beef, Round - Beef, Shanks - Lamb, Lean with Bone - Lamb, Lean without Bone - Pork, Lean Meat (Leg or Loin without Fat) - Pork, Average Meat - Pork, Semi-Fatty - Beef, Ground Special - Beef, Ground Regular - Beef, Fatty with Bone - Beef, Fatty without Bone - Beef, Lean (Without Fat or Bone) - Beef, Semi-Fatty (Steak Type) - Lamb, Fatty Meat - Beef, Shank - Beef, Heart - Pork, Semi-Fatty Ribs - Pork, Shoulder - Beef, Skirt - Beef, Fillet - Beef, Liver - Beef, Stomach and Intestines (Raw) - Beef, Hooves - Pork, Fresh Hooves - Beef, Neck - Beef, Brain - Beef, Tripe - Venison, Lean Raw Meat - Pork, Ground Meat - Cooked Beef - Cooked Pork |
| Processed Meat | - Res, carne seca - Res, cecina oreada - Chicharrón de cerdo (con grasa) - Chorizo de cerdo - Jamón de pavo (rebanada) - Jamón de cerdo (rebanada) - Longaniza de cerdo - Moronga - Mortadela - Queso de puerco - Salami (carne de cerdo y res) - Salchicha de cerdo - Tocino de puerco - Manteca o grasas animales promedio - Cueritos de cerdo - Salchicha de pavo - Bolonia de pavo (embutido) - Jamón endiablado, industrializado - Cerdo, cecina - Cerdo, chuleta ahumada | - Beef, dried meat - Beef, sun-dried salted meat - Pork cracklings (with fat) - Pork sausage - Turkey ham (sliced) - Pork ham (sliced) - Pork longaniza (a type of sausage) - Blood sausage - Mortadella - Pork cheese - Salami (pork and beef) - Pork sausage - Pork bacon - Lard or average animal fats - Pork skin - Turkey sausage - Bologna (turkey, processed) - Devilish ham, industrialized - Pork, sun-dried salted meat - Pork, smoked chop |

# **S4 Table- Categorization of Cuts of Meat in the Mexican National Health and Nutrition Survey, 2016**

| Categorization of Cut of Meat | Original survey items | English translation |
| --- | --- | --- |
| Cheaper Cuts of Meat | - Chorizo de cerdo - Jamón de pavo (rebanada) - Jamón de cerdo (rebanada) - Longaniza de cerdo - Mortadela - Queso de puerco - Salchicha de cerdo - Cueritos de cerdo - Salchicha de pavo - Bolonia de pavo (embutido) - Jamón endiablado, industrializado - Cerdo, chuleta ahumada - Cerdo, carne magra (pierna o lomo sin grasa) - Cerdo, carne promedio - Cerdo, espaldilla - Cerdo, patas frescas - Cerdo, carne molida | - Pork Sausage - Turkey Ham (sliced) - Pork Ham (sliced) - Pork Longaniza (a type of sausage) - Mortadella - Pork Cheese - Pork Sausage - Pork Skin (Pork Rinds) - Turkey Sausage - Bologna (Turkey, Processed) - Devilish Ham - Smoked Pork Chop - Pork, Lean Meat (Leg or Loin without Fat) - Pork, Average Meat - Pork Shoulder - Fresh Pork Hooves - Pork, Ground Meat |
| Expensive Cuts of Meat | - Res, carne seca - Res, cecina oreada - Chicharrón de cerdo (con grasa) - Moronga - Salami (carne de cerdo y res) - Tocino de puerco - Manteca o grasas animales promedio - Cerdo, cecina - Res, aguayón - Res, agujas - Carnero, magra con hueso - Carnero, magra sin hueso - Cerdo, semigrasosa - Res, carne molida especial - Res, carne molida regular - Res, carne grasosa con hueso - Res, carne grasosa sin hueso - Res, carne magra (sin grasa ni hueso) - Res, carne semigrasosa (tipo bistec) - Carnero, carne grasosa - Res, chambarete - Res, corazón - Cerdo, costilla semigrasosa - Res, falda - Res, filete - Res, hígado - Res, estomago e intestinos crudos - Res, patas - Res, pescuezo - Res, sesos - Res, tripas - Venado, carne magra cruda - Carne de res cocida - Carne de cerdo cocida | - Beef, Dried Meat - Beef, Sun-dried Salted Meat - Pork Cracklings (with fat) - Blood Sausage - Salami (Pork and Beef) - Pork Bacon - Lard or Average Animal Fats - Pork, Sun-dried Salted Meat - Beef, Round - Beef, Shanks - Lamb, Lean with Bone - Lamb, Lean without Bone - Pork, Semi-Fatty - Beef, Ground Special - Beef, Ground Regular - Beef, Fatty with Bone - Beef, Fatty without Bone - Beef, Lean (Without Fat or Bone) - Beef, Semi-Fatty (Steak Type) - Lamb, Fatty Meat - Beef, Shank - Beef, Heart - Pork, Semi-Fatty Ribs - Beef, Skirt - Beef, Fillet - Beef, Liver - Beef, Stomach and Intestines (Raw) - Beef, Hooves - Beef, Neck - Pork, Fresh Hooves - Beef, Brain - Beef, Tripe - Venison, Lean Raw Meat - Cooked Beef - Cooked Pork |
| Includes both red and processed meat. Items were grouped to reflect categorization of ENIGH meat expenditures determined by price.  For ENIGH categorization, cheaper cuts of meat were defined as individual items with a price per unit ≤ 114.00 MXN (the weighted national average price per unit across all three survey rounds for red and processed meat combined). Expensive cuts of meat were defined as individual items with a price per unit > 114.00 MXN. | | |

# **S5 Table - Categorization of Food Groups in the Mexican Teacher’s Cohort, 2006-2014**

| Food Group | Original items | English translation |
| --- | --- | --- |
| Dairy | - Leche entera - Leche descremada - Leche semi descremada - Atole con leche - Queso crema - Queso Oaxaca - Queso Manchego - Queso fresco - Otro queso - Crema - Mantequilla - Yogur - Helado de leche - Danonino - Bebida de lactobacilos | - Whole milk - Skim milk - Semi-skimmed milk - Atole with milk - Cream cheese - Oaxaca cheese - Manchego cheese - Fresh cheese - Another cheese - Cream - Butter - Yogurt - Milk ice cream - Danonino – yogurt drink - Lactobacillus drink |
| Discretionary Foods | - Refresco embotellado de cola - Refresco embotellado de sabor - Pan dulce - Pastelito o dona - Gelatina o flan - Bolsa de churritos - Rebanada de pastel - Galleta dulce - Barra de cereal - Caramelo - Tablilla de chocolate | - Bottled cola soda - Bottled flavored soda - Sweet bread roll - Pastry or doughnut - Gelatin or flan - Bag of ultra-processed corn snacks - Slice of cake - Sweet cookie - Cereal bar - Caramel candy - Chocolate bar |
| Grains, roots, and tubers | - Papa - Arroz - Bolillo de telera - Pan blanco - Pan integral - Tortilla de maíz - Tortilla de harina de trigo - Galleta salada - Avena - Sopa de pasta - Cereal de caja - Cereal alto en fibra | - Potato - Rice - White roll - White bread - Whole wheat bread - Corn tortilla - Flour tortilla - Salted cracker - Oatmeal - Pasta soup - Boxed cereal - High-fiber cereal |
| Seafood | - Atún - Sardina - Huachinango - Pescado seco - Camarón - Pulpo, calamar | - Tuna - Sardine - Red snapper - Dried fish - Shrimp - Octupus, squid |
| Poultry | - Pollo | - Chicken |
| Mixed dishes* | - Barbacoa - Birria - Hamburguesa - Hot dog - Pizza - Tacos - Torta de puerco - Sope, quesidilla - Pancita - Pozole - Tamal | - Barbeque - Birria - Hamburger - Hot dog - Pizza - Tacos - Pork sandwich - Sope, quesadilla - Pancita - Pozole - Tamale |
| Fruits and vegetables, | - Plátano - Durazno o chabacano - Manzana - Pera - Naranja o mandarina - Toronja - Uvas - Ciruela - Fresas - Melón - Sandía - Mango - Mamey - Tuna - Zapote - Papaya - Piña - Guayaba - Pasitas - Jícama - Brócoli - Col - Elote - Calabacitas - Lechuga - Verduras (espinacas, acelgas, o quelites) - Jitomate en salsa - Jitomate crudo - Pepino - Tomate verde en salsa - Nopal - Aguacate - Flor de calabaza - Betabel - Cebollitas - Zanahoria | - Banana - Peach or apricot - Apple - Pear - Orange or tangerine - Grapefruit - Grapes - Plums - Strawberries - Melon - Watermelon - Mango - Mamey - Prickly pear - Sapote - Papaya - Pineapple - Guava - Raisins - Jicama - Broccoli - Cabbage - Corn - Zucchini - Lettuce leaf - Vegetables (spinach, Swiss chard, or quelites) - Tomato in sauce - Raw tomato - Cucumber - Green tomato in sauce - Nopal - Avocado - Squash blossoms - Beet - Green onions - Carrots |
| Legumes, nuts and seeds | - Cacahuates naturales - Nueces - Almendras - Leche de soya - Ejotes - Chícharo - Habas verdes - Lentejas o garbanzos - Frijoles - Cacahuates | - Natural peanuts - Walnuts - Almonds - Soy milk - Green beans - Peas - Lima beans - Lentils or chickpeas - Beans - Peanuts |
| Red meat | - Bistec de hígado - Carne de res - Carne de cerdo - Carnitas | - Liver steak - Beef - Pork - Carnitas |
| Processed meat | - Tocino - Salchicha - Jamón de cerdo - Jamón de pavo - Otro embutido - Chorizo - Carne seca - Chicharrón | - Bacon - Sausage - Pork ham - Turkey ham - Another type of processed meat - Chorizo - Dried meat - Pork crackling |
| Items were grouped to reflect categorizations in MNHIES food items.  *Mixed dishes were disaggregated into their respective ingredient components based on a Mexican Teacher’s Cohort recipe file. | | |

# **S6 Table - Sociodemographic characteristics of ENIGH households in Mexico, by survey year and for total sample, 2018-2022**

|  | **Overall**  **n = 248,226** | **2018**  **n = 72,889** | **2020**  **n = 87,130** | **2022**  **n = 88,207** |
| --- | --- | --- | --- | --- |
| **Age of head of the household** | 51.1 (0.1) | 50.1 (0.1) | 51.4 (0.1) | 51.6 (0.1) |
| **Sex of head of the household** | |  |  |  |
| Male | 69.6% | 71.2% | 70.1% | 67.6% |
| **Speaks Indigenous Language** | 7.3% | 7.3% | 7.0% | 7.5% |
| **Adult Equivalent** | 4.6 (0.0) | 4.7 (0.0) | 4.6 (0.0) | 4.5 (0.0) |
| **Education of head of household** | |  |  |  |
| Elementary | 36.3% | 37.7% | 36.6% | 34.8% |
| Middle School | 26.0% | 26.0% | 25.7% | 26.1% |
| High School | 15.3% | 14.5% | 15.2% | 15.9% |
| College | 20.0% | 19.3% | 20.0% | 20.7% |
| Postgraduate | 2.4% | 2.4% | 2.5% | 2.5% |
| **Region of Residence** |  |  |  |  |
| Urban | 77.5% | 77.0% | 78.4% | 77.0% |
| **Trimestral**  **Household Income** | 48,274.7 (278.15) | 44,274.6 (589.03) | 43,972.0 (412.49) | 56,030.0 (439.30) |
| Mean (SE). Other values are percentages as indicated.  Household income is reported as mean (SE) and in Mexican pesos (MXN).  Statistics are weighted taking into account expansion factors and complex survey design.  Sample sizes are unweighted. | | | | |

# **S7 Table– Proportion of Households with Non-Zero Consumption per Food Group in ENIGH by Survey Round and in Total Sample, 2018-2022**

| **Food Category** | **Overall**  **n = 248,226** | | **2018**  **n = 72,889** | **2020**  **n = 87,130** | **2022**  **n = 88,207** | **P-Value*** |
| --- | --- | --- | --- | --- | --- | --- |
| **Dairy** | | 79.1% | 81.0% | 77.9% | 78.5% | **<0.01** |
| **Eggs** | | 66.3% | 66.3% | 65.9% | 66.6% | 0.2 |
| **Discretionary Foods** | | 88.4% | 88.6% | 87.9% | 88.8% | **<0.05** |
| **Fruits and vegetables** | | 86.7% | 86.8% | 87.1% | 86.3% | **<0.01** |
| **Grains, roots, and tubers** | | 96.9% | 97.0% | 96.9% | 96.8% | 0.4 |
| **Processed Meat** | | 54.0% | 54.8% | 52.7% | 54.5% | **<0.001** |
| **Legumes, Nuts, and Seeds** | | 49.3% | 50.5% | 48.8% | 48.6% | <0.001 |
| **Red Meat** | | 59.4% | 57.9% | 60.3% | 59.8% | **<0.001** |
| **Poultry** | | 61.1% | 61.7% | 61.4% | 60.3% | **< 0.05** |
| **Seafood** | | 20.1% | 20.8% | 20.6% | 19.1% | **<0.01** |
| **Other Foods** | | 75.5% | 76.0% | 75.3% | 75.2% | 0.08 |
| Survey-weighted percentages.  *Chi-squared test with Rao & Scott’s second-order correction. Bolded p-values are significant at the alpha = 0.05 level of significance. | | | | | | |

# **S8 Table- Proportion of Households with Non-Zero Consumption per Food Group in ENIGH by Income Quintile, 2018-2022**

| **Food Category** | **Quintile 1**  **n= 49,319** | **Quintile 2 n= 49,707** | **Quintile 3**  **n = 49,766** | **Quintile 4 n =49,769** | **Quintile 5**  **n = 49,665** | **P-Value** |
| --- | --- | --- | --- | --- | --- | --- |
| **Dairy** | 66.6% | 78.6% | 82.3% | 83.8% | 83.0% | **<0.001** |
| **Eggs** | 66.3% | 70.2% | 69.6% | 66.4% | 59.4% | **<0.001** |
| **Discretionary Foods** | 83.9% | 87.0% | 89.4% | 90.6% | 90.6% | **<0.001** |
| **Seafood** | 16.5% | 17.5% | 18.9% | 21.0% | 26.2% | **<0.001** |
| **Fruits and vegetables** | 83.6% | 87.1% | 88.1% | 88.2% | 86.3% | **<0.001** |
| **Grains, roots, and tubers** | 95.6% | 97.2% | 97.9% | 97.8% | 95.8% | **<0.001** |
| **Legumes, nuts and seeds** | 52.8% | 53.0% | 51.2% | 48.4% | 41.6% | **<0.001** |
| **Other foods** | 72.2% | 75.5% | 76.4% | 77.1% | 75.9% | **<0.001** |
| **Poultry** | 50.1% | 61.2% | 64.7% | 65.0% | 63.5% | **<0.001** |
| **Processed Meat** | 38.3% | 52.7% | 59.0% | 60.3% | 58.3% | **<0.001** |
| **Red Meat** | 38.2% | 55.9% | 63.5% | 68.1% | 69.0% | **<0.001** |
| Survey-weighted percentages.  *Chi-squared test with Rao & Scott’s second-order correction. Bolded p-values are significant at the alpha = 0.05 level of significance. | | | | | | |

# **S9 Table– Average per unit value per Food Group in ENIGH by Survey Round and in Total Sample, 2018-2022**

| **Food Category** | **Overall**  **n = 248,226** | **2018**  **n = 72, 89** | **2020**  **n = 87,130** | **2022**  **n = 88,207** | **P-Value*** |
| --- | --- | --- | --- | --- | --- |
| **Dairy** | 44.8 (0.1) | 37.0 (0.2) | 43.4 (0.2) | 53.4 (0.2) | **<0.001** |
| **Eggs** | 37.8 (0.1) | 30.0 (0.1) | 34.9 (0.1) | 47.8 (0.1) | **<0.001** |
| **Discretionary Foods** | 41.2 (0.1) | 34.9 (0.1) | 40.6 (0.1) | 47.6 (0.2) | **<0.001** |
| **Fruits and vegetables** | 26.6 (0.0) | 22.7 (0.1) | 25.0 (0.1) | 31.8 (0.1) | **<0.001** |
| **Grains, roots, and tubers** | 22.4 (0.0) | 18.9 (0.1) | 20.3 (0.0) | 27.6 (0.1) | **<0.001** |
| **Processed Meat** | 98.0 (0.1) | 86.3 (0.2) | 93.7 (0.2) | 112.7 (0.2) | **<0.001** |
| **Red Meat** | 123.7 (0.2) | 108.7 (0.2) | 114.7 (0.2) | 146.1 (0.3) | **<0.001** |
| **Poultry** | 80.9 (0.3) | 70.0 (0.7) | 75.7 (0.5) | 95.8 (0.2) | **<0.001** |
| **Legumes, nuts and seeds** | 36.0 (0.1) | 29.6 (0.1) | 37.0 (0.1) | 40.8 (0.1) | **<0.001** |
| **Seafood** | 115.8 (0.3) | 99.7 (0.4) | 109.7 (0.3) | 136.2 (0.8) | **<0.001** |
| **Other Foods** | 38.1 (0.2) | 33.6 (0.3) | 36.2 (0.3) | 44.1 (0.3) | **<0.001** |
| Survey-weighted average (SE). Unit values were derived by dividing food group expenditure by units purchased. Per unit values are reported in Mexican pesos (MXN).  *Wilcoxon rank-sum test for complex survey samples. Bolded p-values are significant at the alpha = 0.05 level of significance. | | | | | |

# **S10 Table– Budget Share of Total Food Expenditure per Food Group in ENIGH by Survey Round and in Total Sample, 2018-2022**

| **Food Category** | | **Overall**  **n = 248, 226** | | **2018**  **n = 72,889** | | **2020**  **n = 87,130** | | **2022**  **n = 88,207** | | **P-Value*** | | |
| --- | --- | --- | --- | --- | --- | --- | --- | --- | --- | --- | --- | --- |
| **Dairy** | 9.3 (0.0) | | 10.1 (0.1) | | 8.9 (0.1) | | 8.8 (0.1) | | **<0.001** | |  |  |
| **Eggs** | 4.5 (0.0) | | 4.3 (0.0) | | 4.5 (0.0) | | 4.9 (0.0) | | **<0.001** | |  |  |
| **Discretionary Foods** | 19.7 (0.1) | | 19.0 (0.1) | | 20.1 (0.1) | | 20.0 (0.1) | | **<0.01** | |  |  |
| **Fruits and vegetables** | 13.0 (0.0) | | 13.0 (0.1) | | 13.3 (0.1) | | 12.6 (0.1) | | **<0.001** | |  |  |
| **Grains, roots, and tubers** | 17.2 (0.0) | | 17.5 (0.1) | | 16.8 (0.1) | | 17.3 (0.1) | | **<0.001** | |  |  |
| **Processed Meat** | 4.3 (0.0) | | 4.4 (0.0) | | 4.2 (0.0) | | 4.4 (0.0) | | **<0.001** | |  |  |
| **Red Meat** | 11.0 (0.0) | | 10.6 (0.1) | | 11.1 (0.1) | | 11.3 (0.1) | | **<0.001** | |  |  |
| **Poultry** | 9.3 (0.0) | | 9.2 (0.1) | | 9.2 (0.1) | | 9.4 (0.1) | | 0.7 | |  |  |
| **Legumes, nuts, and seeds** | 2.8 (0.0) | | 2.8 (0.0) | | 3.0 (0.0) | | 2.5 (0.0) | | **<0.001** | |  |  |
| **Seafood** | 2.0 (0.0) | | 2.1 (0.0) | | 2.1 (0.0) | | 1.9 (0.0) | | **<0.001** | |  |  |
| **Other Foods** | 6.9 (0.0) | | 7.0 (0.1) | | 6.8 (0.0) | | 6.9 (0.0) | | **<0.05** | |  |  |
| Survey-weighted average share, % (SE). Budget shares were derived by dividing expenditures in food group by total food expenditures.  * Wilcoxon rank-sum test for complex survey samples. Bolded p-values are significant at the alpha = 0.05 level of significance. | | | | | | | | | | | |  |

# **S11 Table– Budget Share of Total Food Expenditure per Food Group in ENIGH by Income Quintile, 2018-2022**

| **Food Category** | **Quintile 1**  **n= 49,319** | **Quintile 2 n= 49,707** | **Quintile 3**  **n = 497,66** | **Quintile 4**  **n =49,769** | **Quintile 5**  **n = 49,665** | **P-Value*** |
| --- | --- | --- | --- | --- | --- | --- |
| **Dairy** | 8.2 (0.1) | 9.1 (0.1) | 9.4 (0.1) | 9.6 (0.1) | 9.9 (0.1) | **<0.001** |
| **Eggs** | 5.7 (0.0) | 5.2 (0.0) | 4.7 (0.0) | 4.1 (0.0) | 3.2 (0.0) | **<0.001** |
| **Discretionary Foods** | 19.3 (0.1) | 18.2 (0.1) | 18.9 (0.1) | 19.7 (0.1) | 22.3 (0.1) | **<0.001** |
| **Seafood** | 1.8 (0.0) | 1.7 (0.0) | 1.8 (0.0) | 2.0 (0.0) | 2.8 (0.1) | **<0.001** |
| **Fruits and vegetables** | 13.1 (0.1) | 12.9 (0.1) | 12.7 (0.1) | 12.8 (0.1) | 13.2 (0.1) | **<0.001** |
| **Grains, roots, and tubers** | 20.7 (0.1) | 18.9 (0.1) | 17.6 (0.1) | 16.2 (0.1) | 13.2 (0.1) | **<0.001** |
| **Other foods** | 8.2 (0.1) | 7.0 (0.1) | 6.5 (0.0) | 6.4 (0.0) | 6.6 (0.1) | **<0.001** |
| **Poultry** | 8.5 (0.1) | 9.6 (0.1) | 9.7 (0.1) | 9.6 (0.1) | 8.8 (0.1) | **<0.001** |
| **Legumes, nuts and seeds** | 4.5 (0.1) | 3.2 (0.0) | 2.6 (0.0) | 2.2 (0.0) | 1.6 (0.0) | **<0.001** |
| **Processed Meat** | 3.4 (0.0) | 4.4 (0.0) | 4.7 (0.0) | 4.7 (0.0) | 4.4 (0.0) | **<0.001** |
| **Red Meat** | 6.7 (0.1) | 9.8 (0.1) | 11.5 (0.1) | 12.8 (0.1) | 13.8 (0.1) | **<0.001** |
| Survey-weighted average share, % (SE). Budget shares were derived by dividing expenditures in food group by total food expenditures.  * Wilcoxon rank-sum test for complex survey samples. Bolded p-values are significant at the alpha = 0.05 level of significance. | | | | | | |

# **S12 Table – Own-price elasticity of demand for red meat and other food groups by year in Mexico, 2018-2022**

| **Food Category** | **Own-price elasticity 2018** | **Own-price elasticity 2020** | **Own-price elasticity 2022** |
| --- | --- | --- | --- |
| **Dairy** | -1.57 (0.01)* | -1.54 (0.02)* | -1.49 (0.02)* |
| **Eggs** | -0.55 (0.06)* | -0.42 (0.07)* | -0.50 (0.06)* |
| **Discretionary Foods** | -1.29 (0.01)* | -1.37 (0.01)* | -1.19 (0.01)* |
| **Seafood** | -2.13 (0.08)* | -2.13 (0.07)* | -1.89 (0.08)* |
| **Fruits and vegetables** | -1.74 (0.02)* | -1.80 (0.02)* | -1.59 (0.02)* |
| **Grains, roots, and tubers** | -1.12 (0.01)* | -1.12 (0.01)* | -1.05 (0.01)* |
| **Legumes, nuts, and seeds** | -1.06 (0.07)* | -0.95 (0.07)* | -0.99 (0.08)* |
| **Poultry** | -0.57 (0.01)* | -0.81 (0.02)* | -0.76 (0.02)* |
| **Processed Meat** | -0.92 (0.07)* | -0.92 (0.07)* | -0.66 (0.06)* |
| **Red Meat** | -0.78 (0.08)* | -0.80 (0.08)* | -0.64 (0.07)* |
| *Significant at 1%. Non-compensated price elasticity standard errors in parentheses.  The ‘Other’ food group category was omitted to avoid collinearity in OLS estimation.  Demand shifters included educational attainment of the head of household, region of residence (urban vs rural), adult equivalent based on estimations derived from the Mexican population. | | | |

# **S13 Table- Cross-price elasticity of demand for red meat and other food groups by year in Mexico, 2018-2022**

| **Food Category** | **Cross-price elasticity with red meat 2018** | **Cross-price elasticity with red meat 2020** | **Cross-price elasticity with red meat 2022** |
| --- | --- | --- | --- |
| **Dairy** | 0.10 (0.02)* | 0.04 (0.02) | 0.02 (0.03) |
| **Eggs** | 0.10 (0.06) | 0.25 (0.06)* | 0.02 (0.05) |
| **Discretionary Foods** | -0.48 (0.01)* | -0.45 (0.01)* | -0.46 (0.01)* |
| **Seafood** | -0.13 (0.12) | -0.57 (0.11)* | -0.63 (0.13)* |
| **Fruits and vegetables** | 0.33 (0.02)* | 0.37 (0.02)* | 0.45 (0.02)* |
| **Grains, roots, and tubers** | 0.30 (0.01)* | 0.32 (0.02)* | 0.31 (0.01)* |
| **Legumes, nuts and seeds** | -0.05 (0.09) | 0.06 (0.08) | -0.11 (0.10) |
| **Poultry** | -0.31 (0.03)* | -0.30 (0.03)* | -0.29 (0.03)* |
| **Processed Meat** | -0.11 (0.06) | -0.10 (0.07) | -0.20 (0.06)* |
| **Red Meat** | - | - | - |
| *Significant at 1%. Non-compensated price elasticity standard errors in parentheses.  The ‘Other’ food group category was omitted to avoid collinearity in OLS estimation.  Demand shifters included educational attainment of the head of household, region of residence (urban vs rural), and adult equivalent based on estimations derived from the Mexican population. | | | |

# **S14 Table - Cross-price elasticity of demand for processed meat and other food groups by year in Mexico, 2018-2022**

| **Food Category** | **Cross-price elasticity with processed meat 2018** | **Cross-price elasticity with processed meat 2020** | **Cross-price elasticity with processed meat 2022** |
| --- | --- | --- | --- |
| **Dairy** | -0.11 (0.01)* | -0.08 (0.01) | -0.13 (0.01)* |
| **Eggs** | -0.28 (0.05) | -0.31 (0.04)* | -0.05 (0.04) |
| **Discretionary Foods** | -0.02 (0.01)* | -0.04 (0.01)* | -0.06 (0.01)* |
| **Seafood** | 0.38 (0.07) | 0.25 (0.06)* | 0.39 (0.07)* |
| **Fruits and vegetables** | 0.07 (0.02)* | 0.04 (0.02)* | -0.04 (0.02)* |
| **Grains, roots, and tubers** | 0.01 (0.01)* | -0.01 (0.01) | 0.05 (0.01)* |
| **Legumes, nuts and seeds** | 0.33 (0.06) | 0.52 (0.05)* | 0.38 (0.07)* |
| **Poultry** | 0.02 (0.01)* | 0.10 (0.02)* | 0.00 (0.02) |
| **Processed Meat** | - | - | - |
| **Red Meat** | -0.05 (0.03)* | -0.04 (0.03)* | -0.08 (0.02)* |
| *Significant at 1%. Non-compensated price elasticity standard errors in parentheses.  The ‘Other’ food group category was omitted to avoid collinearity in OLS estimation.  Demand shifters included educational attainment of the head of household, region of residence (urban vs rural), and adult equivalent based on estimations derived from the Mexican population. | | | |

# **S15 Table - Own- and cross-price elasticity of demand for red and processed meat with other food groups comparing lowest to highest income quintile in Mexico, 2018-2022**

|  | **Cross-price elasticity with red meat** | | **Cross price elasticity with processed meat** | |
| --- | --- | --- | --- | --- |
| **Food Category** | **Low Income** | **High Income** | **Low Income** | **High Income** |
| **Dairy** | 0.17 (0.03)* | 0.07 (0.03) | -0.11 (0.02)* | -0.08 (0.02)* |
| **Eggs** | 0.11 (0.06) | 0.06 (0.10) | -0.28 (0.04)* | -0.08 (0.08) |
| **Discretionary Foods** | -0.40 (0.01)* | -0.47 (0.01)* | 0.02 (0.01) | -0.08 (0.01)* |
| **Seafood** | -0.31 (0.18) | -0.52 (0.13)* | 0.32 (0.09)* | 0.25 (0.08)* |
| **Fruits and vegetables** | 0.37 (0.03)* | 0.30 (0.03)* | 0.08 (0.02)* | -0.05 (0.02) |
| **Grains, roots, and tubers** | 0.31 (0.02)* | 0.34 (0.03)* | -0.03 (0.01)* | 0.06 (0.02)* |
| **Legumes, nuts and seeds** | 0.02 (0.07) | -0.03 (0.20) | 0.20 (0.05)* | 0.24 (0.13) |
| **Poultry** | -0.42 (0.04)* | -0.18 (0.04)* | 0.01 (0.02) | 0.05 (0.02) |
| **Processed Meat** | -0.08 (0.10) | -0.25 (0.09)* | - | - |
| **Red Meat** | - | - | -0.04 (0.05) | -0.08 (0.03)* |

*Significant at 1%. Non-compensated price elasticity standard errors in parentheses.

The ‘Other’ food group category was omitted to avoid collinearity in OLS estimation.

Demand shifters included educational attainment of the head of household, year of survey (2018, 2020, and 2022), region of residence (urban vs rural), adult equivalent based on estimations derived from the Mexican population.

# **S16 Table – Own- and cross-price elasticity of demand for cuts of meat and other food groups in Mexico, 2018-2022**

| **Food Category** | **Own-price elasticity** | **Cross-price elasticity with cheaper meat** | **Cross-price elasticity with expensive meat** |
| --- | --- | --- | --- |
| **Dairy** | -1.58 (0.01)* | 0.09 (0.01)* | -0.07 (0.02)* |
| **Eggs** | -0.44 (0.04)* | -0.52 (0.03)* | 0.27 (0.04)* |
| **Discretionary Foods** | -1.30 (0.00)* | -0.03 (0.00)* | -0.35 (0.01)* |
| **Seafood** | -2.05 (0.05)* | 0.55 (0.04)* | -0.82 (0.08)* |
| **Fruits and vegetables** | -1.74 (0.01)* | -0.06 (0.01)* | 0.42 (0.01)* |
| **Grains, roots, and tubers** | -1.02 (0.01)* | 0.03 (0.01)* | 0.26 (0.01)* |
| **Legumes, nuts, and seeds** | -0.88 (0.04)* | 0.33 (0.04)* | 0.07 (0.06) |
| **Poultry** | -0.71 (0.01)* | 0.09 (0.01)* | -0.17 (0.02)* |
| **Cheaper cuts of Meat** | -0.83 (0.03)* | - | -0.27 (0.03)* |
| **Expensive cuts of Meat** | -0.78 (0.06)* | -0.19 (0.02)* | - |
| *Significant at 1%. Non-compensated price elasticity standard errors in parentheses.  The ‘Other’ food group category was omitted to avoid collinearity in OLS estimation.  Demand shifters included educational attainment of the head of household, year of survey (2018, 2020, and 2022), region of residence (urban vs rural), adult equivalent based on estimations derived from the Mexican population. | | | |

# **S2 Figure – Own-price elasticity of demand for expensive and cheap cuts of meat by income quintile in Mexico, 2018-2022**

**
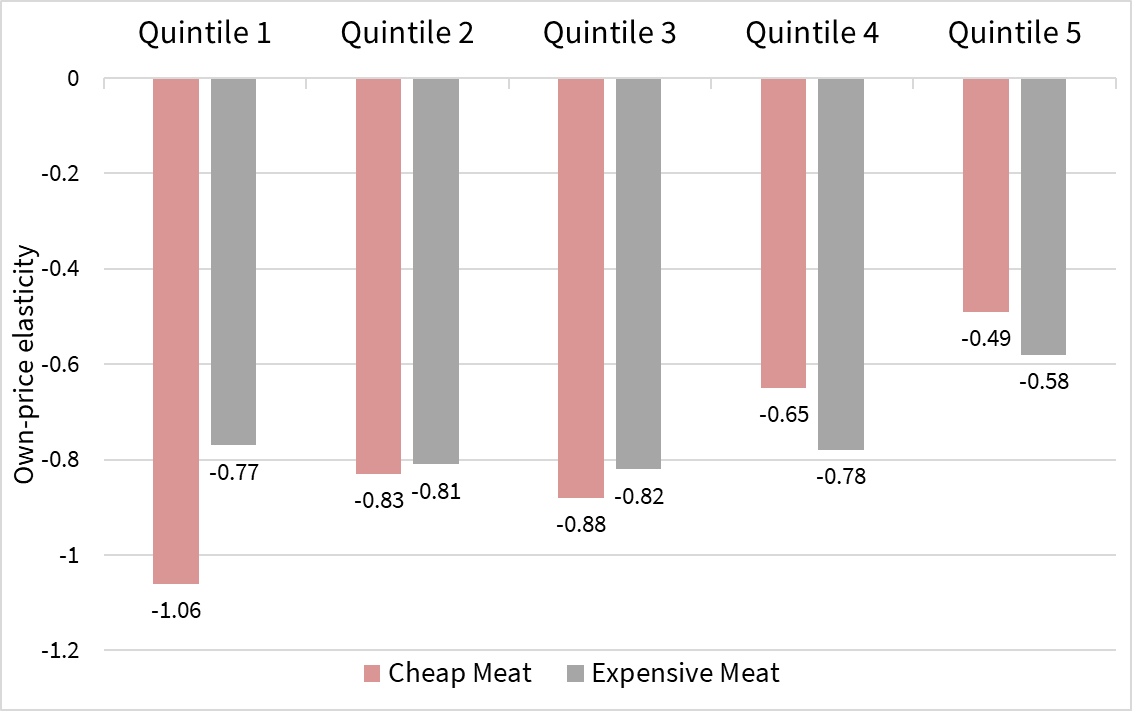
**

All estimations are significant at 1%.

The ‘Other’ food group category was omitted to avoid collinearity in OLS estimation.

Demand shifters included educational attainment of the head of household, year of survey (2018, 2020, and 2022), region of residence (urban vs rural), and adult equivalent based on estimations derived from the Mexican population.

# **S17 Table- Own- and cross-price elasticity of demand for cuts of meat and other food groups comparing lowest to highest income quintile in Mexico, 2018-2022**

|  | **Cross-price elasticity with cheaper meat** | | **Cross price elasticity with expensive meat** | |
| --- | --- | --- | --- | --- |
| **Food Category** | **Low Income** | **High Income** | **Low Income** | **High Income** |
| **Dairy** | 0.03 (0.02) | 0.15 (0.02)* | 0.02 (0.04) | -0.07 (0.04) |
| **Eggs** | -0.45 (0.05)* | -0.54 (0.09)* | 0.21 (0.06)* | 0.34 (0.13)* |
| **Discretionary Foods** | 0.02 (0.01) | -0.03 (0.01)* | -0.29 (0.02)* | -0.33 (0.02)* |
| **Seafood** | 0.62 (0.10)* | 0.25 (0.08)* | -1.03 (0.19)* | -0.66 (0.14)* |
| **Fruits and vegetables** | 0.01 (0.02) | -0.07 (0.03)* | 0.42 (0.03)* | 0.23 (0.04)* |
| **Grains, roots, and tubers** | 0.04 (0.01)* | -0.03 (0.02) | 0.16 (0.02)* | 0.44 (0.03)* |
| **Legumes, Nuts, and Seeds** | 0.19 (0.05)* | -0.04 (0.14) | 0.03 (0.08) | 0.02 (0.23) |
| **Poultry** | 0.02 (0.03) | 0.15 (0.02)* | -0.29 (0.04)* | -0.16 (0.04)* |
| **Cheaper cuts of Meat** | - | - | -0.24 (0.08)* | -0.47 (0.07)* |
| **Expensive cuts of Meat** | -0.19 (0.08)* | -0.26 (0.04)* | - | - |

*Significant at 1%. Non-compensated price elasticity standard errors in parentheses.

The ‘Other’ food group category was omitted to avoid collinearity in OLS estimation.

Demand shifters included educational attainment of the head of household, year of survey (2018, 2020, and 2022), region of residence (urban vs rural), adult equivalent based on estimations derived from the Mexican population.

# **S18 Table – Own- and cross-price elasticities of demand for red and processed meat and other food groups in Mexico, 2018-2022**

| **Food Category** | **Own-price elasticity** | **Cross-price elasticity with red meat** | **Cross-price elasticity with processed meat** |
| --- | --- | --- | --- |
| **Dairy** | -1.50 (0.01)* | -0.16 (0.01)* | -0.08 (0.01)* |
| **Eggs** | -0.54 (0.02)* | -0.27 (0.02)* | -0.14 (0.01)* |
| **Discretionary Foods** | -1.37 (0.01)* | -0.14 (0.01)* | 0.00 (0.00) |
| **Seafood** | -2.14 (0.03)* | -0.13 (0.03)* | 0.29 (0.02)* |
| **Fruits and Vegetables,** | -1.72 (0.01)* | 0.22 (0.01)* | -0.05 (0.01)* |
| **Grains, roots, and tubers** | -0.90 (0.01)* | 0.12 (0.01)* | 0.07 (0.00)* |
| **Legumes, nuts, and seeds** | -0.97 (0.02)* | -0.14 (0.02)* | 0.20 (0.02)* |
| **Other foods** | -1.20 (0.00)* | -0.04 (0.01)* | 0.01 (0.00)* |
| **Poultry** | -0.75 (0.01)* | -0.13 (0.01)* | -0.06 (0.01)* |
| **Processed Meat** | -0.75 (0.02)* | -0.22 (0.02)* | - |
| **Red Meat** | -0.61 (0.02)* | - | -0.10 (0.01)* |
| *Significant at 1%. Non-compensated price elasticity standard errors in parentheses.  The demand system was estimated using Seemingly Unrelated Regression.  Demand shifters included educational attainment of the head of household, year of survey (2018, 2020, and 2022), region of residence (urban vs rural), adult equivalent based on estimations derived from the Mexican population. | | | |

# **S19 Table– Average daily consumption (g) of red and processed meat in ENSANUT 2016 for adult population (≥18 years), by consumption status, and by income quintile**

|  | **Red Meat** | **Processed Meat** | **Animal-source protein foods *** | **Dairy** | **Egg** | **Seafood** | **Legumes, nuts and seeds** | **Poultry** |
| --- | --- | --- | --- | --- | --- | --- | --- | --- |
| **Adult Population ≥18 years of age** | 45.8 (4.9) | 22.4 (3.1) | 132.3 (6.9) | 141.8 (10.5) | 32.4 (2.5) | 11.9 (2.7) | 25.5 (2.0) | 42.1 (4.7) |
| **Income Quintiles** |  |  |  |  |  |  |  |  |
| Q1 | 26.3 (5.4) | 11.2 (4.5) | 100.2 (12.6) | 70.6 (11.1) | 35.6 (6.5) | 12.7 (7.5) | 43.4 (8.9) | 25.7 (6.2) |
| Q2 | 34.3 (5.9) | 18.0 (4.8) | 131.0 (9.8) | 94.0 (11.9) | 40.2 (6.2) | 6.9 (2.4) | 22.7 (3.3) | 49.6 (11.8) |
| Q3 | 51.5 (9.9) | 20.7 (4.6) | 139.1 (16.2) | 101.9 (13.1) | 35.9 (5.5) | 5.9 (2.2) | 25.7 (4.1) | 45.8 (8.2) |
| Q4 | 37.4 (6.7) | 19.5 (4.6) | 119.7 (15.2) | 159.7 (19.5) | 34.8 (6.9) | 11.4 (4.7) | 25.4 (3.9) | 36.1 (14.0) |
| Q5 | 56.2 (9.7) | 28.2 (5.9) | 144.1 (12.4) | 176.8 (20.7) | 27.4 (3.8) | 15.1 (5.3) | 21.9 (3.2) | 45.5 (7.0) |
| **Consumption Status**** |  |  |  |  |  |  |  |  |
| Red Meat Consumers | 101.2 (9.6) | 16.0 (2.6) | 162.4 (12.8) | 134.6 (17.2) | 22.5 (3.1) | 7.6 (2.6) | 22.4 (3.2) | 31.1 (6.1) |
| Processed Meat Consumers | 43.0 (11.8) | 70.6 (7.2) | 121.0 (11.0) | 153.0 (13.8) | 37.5 (5.2) | 10.7 (5.9) | 22.0 (9.2) | 29.9 (7.2) |

Values are mean (SE) grams consumed per day per capita and are weighted to account for complex survey design. Frequencies are weighted to account for complex survey design.

*Animal-source protein foods is the sum of red meat, poultry, eggs, and fish consumption as per recommended 84/day for EAT-Lancet and 140g/day for 2023 Mexican Dietary Guidelines.

** Red meat consumers are those that reported non-zero single-day red meat intake in the 24-hr dietary recall. Processed meat consumers are those that reported non-zero single-day red meat intake in the 24-hr dietary recall.

## **S20 Table - Correlations between changes in Red, Processed, and Total Meat consumption with Non-Meat Food Groups for Period 1 (2006 -2008) and Period 2 (2008 – 2014) in 1,417 Mexican Women from the Mexican Teachers’ Cohort**

|  | **Period 1** |  | **Period 2** |  |
| --- | --- | --- | --- | --- |
|  | **Estimate (95% CI)** | **P-value** | **Estimate (95% CI)** | **P-value** |
| **Grains, roots, and tubers** | | | | |
| **Red Meat** |  |  |  |  |
| Intercept | -30.29 (-32.12, -28.46) | <0.001 | -1.46 (-2.23, -0.69) | <0.001 |
| Grains | 0.00 (-0.02, 0.02) | 0.91 | -0.01 (-0.02, 0.01) | 0.21 |
| Energy | 0.01 (0.01, 0.01) | <0.001 | 0.01 (0.01, 0.01) | <0.001 |
| **Processed Meat** |  |  |  |  |
| Intercept | -10.75 (-11.80, -9.70) | <0.001 | -0.59 (-1.00, -0.17) | <0.01 |
| Grains | **0.01 (0.00, 0.02)** | **<0.05** | -0.00 (-0.01, 0.01) | 0.73 |
| Energy | 0.00 (0.00, 0.01) | <0.001 | 0.00 (0.00, 0.00) | <0.001 |
| **Total Meat** |  |  |  |  |
| Intercept | -41.04 (-43.31, -38.78) | <0.001 | -2.04 (-2.99, -1.10) | <0.001 |
| Whole grain | 0.01 (-0.01, 0.04) | 0.3 | -0.01 (-0.03, 0.01) | 0.24 |
| Energy | 0.02 (0.01, 0.02) | <0.001 | 0.01 (0.01, 0.01) | <0.001 |
| **Discretionary Food** | | | | |
| **Red Meat** |  |  |  |  |
| Intercept | -29.66 (-31.10, -28.23) | <0.001 | -1.34 (-2.11, -0.57) | <0.001 |
| Discretionary Food | 0.01 (-0.00, 0.01) | 0.07 | 0.01 (-0.00, 0.01) | 0.1 |
| Energy | 0.01 (0.01, 0.01) | <0.001 | 0.01 (0.01, 0.01) | <0.001 |
| **Processed Meat** |  |  |  |  |
| Intercept | -11.61 (-12.43, -10.79) | <0.001 | -0.57 (-0.99, -0.15) | <0.01 |
| Discretionary Food | -0.00 (-0.00, 0.00) | 0.82 | 0.00 (-0.00, 0.00) | 0.75 |
| Energy | 0.01 (0.00, 0.01) | <0.001 | 0.00 (0.00, 0.00) | <0.001 |
| **Total Meat** |  |  |  |  |
| Intercept | -41.28 (-43.05, -39.51) | <0.001 | -1.92 (-2.86, -0.97) | <0.001 |
| Discretionary Food | 0.01 (-0.00, 0.01) | 0.17 | 0.01 (-0.00, 0.01) | 0.14 |
| Energy | 0.02 (0.01, 0.02) | <0.001 | 0.01 (0.01, 0.01) | <0.001 |
| **Dairy** | | | | |
| **Red Meat** |  |  |  |  |
| Intercept | -30.65 (-31.96, -29.35) | <0.001 | -1.47 (-2.25, -0.70) | <0.001 |
| Dairy | -0.00 (-0.01, 0.00) | 0.26 | -0.00 (-0.01, 0.00) | 0.41 |
| Energy | 0.01 (0.01, 0.01) | <0.001 | 0.01 (0.01, 0.01) | <0.001 |
| **Processed Meat** |  |  |  |  |
| Intercept | -11.28 (-12.03, -10.53) | <0.001 | -0.62 (-1.04, -0.19) | <0.01 |
| Dairy | 0.00 (0.00, 0.01) | 0.05 | -0.00 (-0.00, 0.00) | 0.32 |
| Energy | 0.01 (0.00, 0.01) | <0.001 | 0.00 (0.00, 0.00) | <0.001 |
| **Total Meat** |  |  |  |  |
| Intercept | -41.93 (-43.55, -40.31) | <0.001 | -2.09 (-3.05, -1.13) | <0.001 |
| Dairy | 0.00 (-0.01, 0.01) | 0.99 | -0.00 (-0.01, 0.00) | 0.27 |
| Energy | 0.02 (0.01, 0.02) | <0.001 | 0.01 (0.01, 0.01) | <0.001 |

Bolded p-values are significant at the alpha = 0.05 significance level.
Generalized linear models estimated the change in consumed grams/day between periods for red, processed, and total meat by changes in food group (g/day) consumed for the same period adjusting for changes in total daily energy intake.

# **S20 Table (cont.) - Correlations between changes in Red, Processed, and Total Meat consumption with Non-Meat Food Groups for Period 1 (2006 -2008) and Period 2 (2008 – 2014) in 1,417 Mexican Women from the Mexican Teachers’ Cohort**

|  | **Period 1** |  | **Period 2** |  |  |
| --- | --- | --- | --- | --- | --- |
|  | **Estimate (95% CI)** | **P-Value** | **Estimate (95% CI)** | **P-Value** |  |
| **Fruits and vegetables** | | | | | |
| **Red Meat** |  |  |  |  |  |
| Intercept | -32.63 (-34.26, -31.00) | <0.001 | -1.13 (-1.89, -0.37) | <0.01 |  |
| Fruit/Veg | **-0.01 (-0.01, -0.00)** | **<0.001** | **-0.01 (-0.01, -0.01)** | **<0.001** |  |
| Energy | 0.01 (0.01, 0.02) | <0.001 | 0.01 (0.01, 0.01) | <0.001 |  |
| **Processed Meat** |  |  |  |  |  |
| Intercept | -12.34 (-13.28, -11.41) | <0.001 | -0.43 (-0.84, -0.01) | 0.04 |  |
| Fruit/Veg | **-0.00 (-0.00, -0.00)** | **0.01** | **-0.01 (-0.01, -0.00)** | **<0.001** |  |
| Energy | 0.01 (0.00, 0.01) | <0.001 | 0.00 (0.00, 0.01) | <0.001 |  |
| **Total Meat** |  |  |  |  |  |
| Intercept | -44.97 (-46.98, -42.96) | <0.001 | -1.56 (-2.49, -0.63) | <0.001 |  |
| Fruit/Veg | **-0.01 (-0.01, -0.01)** | **<0.001** | **-0.02 (-0.02, -0.01)** | **<0.001** |  |
| Energy | 0.02 (0.02, 0.02) | <0.001 | 0.01 (0.01, 0.02) | <0.001 |  |
|  | **Legumes, nuts, and seeds** | | | |  |
| **Red Meat** |  |  |  |  |  |
| Intercept | -30.45 (-31.81, -29.10) | <0.001 | -1.37 (-2.14, -0.61) | <0.001 |  |
| Legumes | -0.00 (-0.02, 0.01) | 0.8 | **-0.02 (-0.03, -0.01)** | **<0.01** |  |
| Energy | 0.01 (0.01, 0.01) | <0.001 | 0.01 (0.01, 0.01) | <0.001 |  |
| **Processed Meat** |  |  |  |  |  |
| Intercept | -11.74 (-12.51, -10.96) | <0.001 | -0.56 (-0.97, -0.14) | <0.01 |  |
| Legumes | -0.00 (-0.01, 0.00) | 0.33 | **-0.01 (-0.02, -0.00)** | **<0.01** |  |
| Energy | 0.01 (0.00, 0.01) | <0.001 | 0.00 (0.00, 0.00) | <0.001 |  |
| **Total Meat** |  |  |  |  |  |
| Intercept | -42.19 (-43.87, -40.51) | <0.001 | -1.93 (-2.87, -0.99) | <0.001 |  |
| Legumes | -0.01 (-0.02, 0.01) | 0.51 | **-0.03 (-0.04, -0.01)** | **<0.001** |  |
| Energy | 0.02 (0.01, 0.02) | <0.001 | 0.01 (0.01, 0.01) | <0.001 |  |
|  | **Poultry** | | | |  |
| **Red Meat** |  |  |  |  |  |
| Intercept | -29.72 (-31.22, -28.23) | <0.001 | -1.44 (-2.20, -0.67) | <0.001 |  |
| Poultry | 0.04 (-0.01, 0.10) | 0.14 | 0.04 (-0.01, 0.09) | 0.11 |  |
| Energy | 0.01 (0.01, 0.01) | <0.001 | 0.01 (0.01, 0.01) | <0.001 |  |
| **Processed Meat** |  |  |  |  |  |
| Intercept | -10.99 (-11.84, -10.13) | <0.001 | -0.60 (-1.02, -0.19) | <0.01 |  |
| Poultry | **0.04 (0.00, 0.07)** | **<0.05** | **0.05 (0.02, 0.07)** | **<0.01** |  |
| Energy | 0.01 (0.00, 0.01) | <0.001 | 0.00 (0.00, 0.00) | <0.001 |  |
| **Total Meat** |  |  |  |  |  |
| Intercept | -40.71 (-42.55, -38.87) | <0.001 | -2.04 (-2.98, -1.10) | <0.001 |  |
| Poultry | **0.08 (0.01, 0.15)** | **<0.05** | **0.09 (0.03, 0.15)** | **<0.01** |  |
| Energy | 0.02 (0.01, 0.02) | <0.001 | 0.01 (0.01, 0.01) | <0.001 |  |
| Bolded p-values are significant at the alpha = 0.05 significance level. Generalized linear models estimated the change in consumed grams/day between periods for red, processed, and total meat by changes in food group (g/day) consumed for the same period adjusting for changes in total daily energy intake. | | | | |  |

# **S20 Table (cont.) - Correlations between changes in Red, Processed, and Total Meat consumption with Non-Meat Food Groups for Period 1 (2006 -2008) and Period 2 (2008 – 2014) in 1,417 Mexican Women from the Mexican Teachers’ Cohort**

|  | **Period 1** |  | **Period 2** |  |
| --- | --- | --- | --- | --- |
|  | **Estimate (95% CI)** | **P-Value** | **Estimate (95% CI)** | **P-Value** |
| **Seafood** | | | | |
| **Red Meat** |  |  |  |  |
| Intercept | -29.82 (-31.38, -28.25) | <0.001 | -1.46 (-2.23, -0.70) | <0.001 |
| Seafood | 0.03 (-0.02, 0.09) | 0.27 | **0.07 (0.02, 0.12)** | **<0.01** |
| Energy | 0.01 (0.01, 0.01) | <0.001 | 0.01 (0.01, 0.01) | <0.001 |
| **Processed Meat** |  |  |  |  |
| Intercept | -10.75 (-11.64, -9.85) | <0.001 | -0.68 (-1.08, -0.28) | <0.001 |
| Seafood | **0.05 (0.01, 0.08)** | **<0.01** | **0.14 (0.11, 0.17)** | **<0.001** |
| Energy | 0.01 (0.00, 0.01) | <0.001 | 0.00 (0.00, 0.00) | <0.001 |
| **Total Meat** |  |  |  |  |
| Intercept | -40.56 (-42.50, -38.63) | <0.001 | -2.14 (-3.07, -1.21) | <0.001 |
| Seafood | **0.08 (0.01, 0.15)** | **<0.05** | **0.21 (0.15, 0.27)** | **<0.001** |
| Energy | 0.02 (0.01, 0.02) | <0.001 | 0.01 (0.01, 0.01) | <0.001 |
| **Processed Meat** | | | | |
| **Red Meat** |  |  |  |  |
| Intercept | -8.52 (-9.65, -7.40) | <0.001 | -0.42 (-0.83, -0.02) | 0.04 |
| Processed Meat | **0.10 (0.07, 0.13)** | **<0.001** | **0.11 (0.08, 0.14)** | **<0.001** |
| Energy | 0.00 (0.00, 0.01) | <0.001 | 0.00 (0.00, 0.00) | <0.001 |
| **Red Meat** | | | | |
| **Processed Meat** |  |  |  |  |
| Intercept | -26.84 (-28.43, -25.26) | <0.001 | -1.20 (-1.95, -0.45) | <0.01 |
| Red Meat | **0.31 (0.22, 0.39)** | **<0.001** | **0.37 (0.28, 0.47)** | **<0.001** |
| Energy | 0.01 (0.01, 0.01) | <0.001 | 0.01 (0.00, 0.01) | <0.001 |

Bolded p-values are significant at the alpha = 0.05 significance level.
Generalized linear models estimated the change in consumed grams/day between periods for red, processed, and total meat by changes in food group (g/day) consumed for the same period adjusting for changes in total daily energy intake.
