# Supplementary material for: Taxes to red and processed meat to promote sustainable and healthy diets in Mexico
Source: PLoS One. 2025 Jun 27;20(6):e0326616. doi: 10.1371/journal.pone.0326616 (PMC12204545; doi:10.1371/journal.pone.0326616)
Supplement: S1 Text — (DOCX) [file pone.0326616.s002.docx]

# **S1 Text. Supplementary Methods**

Information for all three surveys from the Mexican National Income and Expenditure Survey, known as the *Encuesta Nacional de Ingresos y Gastos de los Hogares* (ENIGH), were administered during the same dates (21^st^ of August to 28^th^ of November) for their respective years to control for seasonality.

Households that did not report food expenditures or that only reported expenditures not directly related to food such as alcohol, tobacco, food preparation, pet food, and non-descript meal events eaten outside the home (i.e, food dispensaries, breakfast, lunch, or dinner) were excluded (orginal sample sizes were n = 73,405 for 2018, n = 87,754 for 2020, and n = 88,823 for 2022). The unweighted sample for each year was n=72,889 in 2018, n=87,130 in 2020, and n=88,207 in 2022.

The model estimates obtained in equation (1) ($\beta_{gj} and \bar{w_{g}} )$ were used to calculate uncompensated Marshallian own- and cross-price elasticities of demand based on observed shares with the following formula:

1. $\varepsilon$ = - $\delta_{jg}$ + $\frac{\hat{\gamma}}{\bar{w_{g}}}$ - $\frac{\hat{\beta}}{\bar{w_{g}}}$ * $\bar{w_{g}}$

As defined, $\varepsilon$ represents the price elasticity of the food group. When *j=g* then $\delta_{jg}$ = 1 indicating own-price elasticity, and otherwise $\delta_{jg}$ = 0 indicating cross-price elasticity [5]. Accordingly, the formulas for own- and cross-price elasticities can be simplified to:

1. $\varepsilon$ = - 1 + $\frac{\hat{\gamma}}{\bar{w_{g}}}$ - $\hat{\beta}$


$$\mathrm{and}$$

2. $\varepsilon$ = $\frac{\hat{\gamma}}{\bar{w_{g}}}$ - $\frac{\hat{\beta}}{\bar{w_{g}}}$ * $\bar{w_{g}}$

We included adult equivalent among the demand shifters since it has been previously included in food demand system analyses for Mexico[3] to account for how household composition may impact welfare due to household composition and the distribution of income amongst members.

*Price Increase Scenarios*

There are multiple combinations of price increases to red and processed meat that would achieve dietary targets (**S1 Figure**). However, we tested price increase scenarios that were previously modelled in other settings.

*Sensitivity Analysis*

‘Expensive cuts’ were classified as those priced above the weighted national average price per unit of meat across all three years [mean (SE) =114·00 (48·4) Mexican Pesos (MXN)]. ‘Cheaper cuts’ were those equal to or below the national average (see list of specific cuts in **S2** **Table**). Price elasticities of expensive and cheaper cuts were estimated using the same demand model.

*Application of demand response to national consumption data*

We calculated average daily intake of each food group for the Mexican adult population and subgroups (i.e., red meat consumers, processed meat consumers, lowest income, highest income) while accounting for the complex survey design using the “survey” package in R.

The Mexican Dietary Guidelines and EAT-Lancet also were designed to allow for substitutions across animal-source protein foods. Namely, these are: red meat, poultry, eggs, and fish with combined recommended daily intake as 140 g/day for the Mexican Dietary Guidelines 2023 and 84 g/day for EAT-Lancet. Therefore, we calculated baseline consumption of each of these food groups and summed them per participant to estimate baseline consumption of animal-source protein foods in the adult population and by subgroup. To calculate consumption of animal-source proteins after price increase, we multiplied percentage change in quantity demanded to baseline consumption of each food group and then summed to obtain average consumption of animal-source protein foods after the price increase

The, we estimated changes in daily intake in the Mexican adult population according to price increase scenarios by multiplying baseline consumption by percent change in quantity demanded according to each price increase scenario.

**Longitudinal analysis of dietary substitutes and complements in cohort of Mexican women**

We conducted a longitudinal analysis in Mexican women from the Mexican Teachers’ Cohort (MTC), a prospective cohort study of 115,314 female teachers across Mexico that initiated in 2006 and 2008[6]. Baseline data for 2006 was only available for teachers residing in the states of Jalisco and Veracruz. For survey rounds where dietary data was collected in 2006, 2008, and 2014, respondents answered questionnaires on dietary intake, sociodemographic characteristics, and medical history. Of the original baseline sample in 2006 consisting of women residing in the states of either Jalisco or Veracruz (n = 27, 979), those who did not complete at least 70 of the 140-item food frequency questionnaires (n = 5,649), those with implausible caloric intake (≤ 500 kcal/day or ≥ 3,500 kcal/day) (n = 3,961), and those who were lost to follow-up (n = 18, 369). The final analytic sample was 1,417 participants.

***Dietary Intake***

MTC evaluated dietary intake using a validated 140-item food frequency questionnaire(FFQ)[7]. The participants responded to questions regarding, on average, how frequently they consumed a specified unit or portion size during the past year. Possible frequencies ranged from never to ≥ 6 servings per day. The FFQ used was previously validated with four 4-day 24-hour recalls and two FFQs in 134 females residing in Mexico City in a 12 month study[7].

We converted FFQ frequency of consumption of each food item to servings per day and then to grams per day using predefined portion sizes. Mixed dishes were disaggregated into their respective ingredients using a recipe file. We converted frequencies into grams consumed per day for eight food groups that reflected those created for the ENIGH expenditure data: 1) dairy, 2) discretionary food, 3) seafood, 4) fruits and vegetables (F&V), 5) legumes, nuts, and seeds (herein, legumes), 6) grains, roots and tubers (herein, grains), 7) poultry, 8) processed meat (including processed red meat and poultry), and 9) unprocessed red meat and offal (herein, red meat) (**S5** **Table**).

Total energy intake was calculated as the product of the specified predefined portion size of each food item and the USDA food-composition table [8] supplemented with a database used in the National Health and Nutrition Survey in Mexico (personal communication).

***Descriptive variables***

The baseline questionnaire collected self-reported information on age, education, ethnicity, and anthropometric measurements. Information on educational attainment (last completed degree) came from the 2008 questionnaire, as it was not collected at baseline. Ethnicity was recorded as the participant having an indigenous background based on whether the participant or her parents spoke an indigenous language. Body-mass index (BMI) was calculated as the weight in kilograms over height in meters squared.

**Statistical Analysis**

The median value was imputed for covariates with missing information, namely: education (7.0%), and BMI (6.7.7%). For indigenous background (meaning those who reported speaking an indigenous language), missing values (0.4%) were assumed to be a “no”.

We calculated total meat as the sum of both unprocessed red and processed meat intake. To describe changes in consumption over the study period, we created variables measuring the change in consumption (grams/day) for each respective period (Period 1: 2006 – 2008 and Period 2: 2008 – 2014) for all meat and non-meat food groups. We also calculated changes in total energy intake for both period 1 and period 2. If there was a change in consumption by ± 0.5 grams/day between each time point, we descriptively categorized them as: 1) no change, 2) decrease, 3) increase, 4) mixed to be reported in-line along with sociodemographic characteristics.

Paired t-tests were used to evaluate whether reported unprocessed red, processed, and total meat consumption (grams/day) were equal to each other between 2006 and 2008, 2008 and 2014, and 2006 and 2014.

To evaluate potential dietary substitution behavior, we applied a generalized linear model with an identity link function to predict changes in unprocessed red, processed, and total meat intake (grams/day) with changes in non-meat food groups (grams/day) adjusting for changes in total energy intake for that period. We considered dietary substitution or complement behavior if a statistically significant correlation was observed in both periods. A positive correlation indicated a dietary complement, and a negative correlation indicated a dietary substitution.
