# Supplementary material for: Taxes to red and processed meat to promote sustainable and healthy diets in Mexico
Source: PLoS One. 2025 Jun 27;20(6):e0326616. doi: 10.1371/journal.pone.0326616 (PMC12204545; doi:10.1371/journal.pone.0326616)
Supplement: S2 Text — (DOCX) [file pone.0326616.s003.docx]

# **S2 Text. Supplemental Results**

*Distribution of sociodemographic characteristics, budget shares, non-zero food expenditures, and prices*

The weighted distribution of sociodemographic characteristics in the ENIGH analytical sample are represented in **S6** **Table**. The weighted proportion of households that reported non-zero food expenditures for each food group is presented in **S7** **Table**. The food groups with the highest proportion of non-zero food expenditure in the pooled sample were ‘grains, roots, and tubers’ (96.9%), discretionary foods (88.4%), and F&V (86.7%). The food groups with the lowest non-zero food expenditure were seafood (20.1%), processed meat (54.0%), and ‘legumes, nuts and seeds’ (54.0%). Differences in households reporting non-zero food expenditure per food group for each survey year were statistically different for all food groups except eggs, ‘grains, roots and tubers’, and other foods. Albeit statistically significant, these differences were small (**S7** **Table**). The proportion of households that reported non-zero consumption stratified by income were statistically different for each food group. Households with the lowest income were less likely to report expenditures on red and processed meat (38.2% and 38.3%, respectively) compared to highest income households (69.0% and 58.3%) (**S8** **Table**). Households in the lowest income quintile reported higher consumption of eggs (66.3% vs 59.4%) and ‘legumes, nuts, and seeds’ (52.8% vs 41.6%) compared to those in the highest. Whereas households in the highest income group reported higher consumption of dairy, discretionary foods, poultry, and seafood compared to the lowest.

In **S9 Table**, average price per unit values (SE) of each food group is presented by year and pooled. Over time, the prices of every food group increased, and these differences were statistically significant across survey years. The most expensive food groups were seafood, red meat, and processed meat per unit. Next, the budget share of each food group out of total food expenditures is illustrated in **S10 Table**. The food groups that constituted the highest budget share overall were discretionary foods (19.7%), ‘grains, roots, and tubers (17.2%), and F&V (13.0 %) whereas the lowest were seafood (2.0 %) and processed meat (4.3%). Differences in budget share were statistically significant across years for each food group except for poultry (**S10 Table**). Additionally, the budget share of each food group stratified by income was statistically significant for all food groups (**S11 Table**). In the lowest income quintile, red and processed meat represented a lower budget share of total food expenditures (6.7% and 3.4%) compared to the highest income quintile (13.8% and 4.4%). ‘Grains, roots, and tubers’, ‘legumes, nuts and seeds’, and eggs represented greater budget shares in the lowest income group than they did in the highest (**S11 Table**).

*Stratified own- and cross-price elasticities*

Both red and processed meat become more elastic whereas poultry became more inelastic over time (**S13** **Table**). Red and processed meat became stronger complements to each other over time (**S13 Tables and S14**). F&V was a stronger substitute to red meat over time and seafood became a stronger complement (**S13 Table**). For processed meat, eggs became a weaker complement and F&V became a complement rather than substitute over time (**S14 Table**).

*Consumption of red and processed meat with other protein rich food groups in Mexican adult population*

The survey-weighted average single-day intake for red and processed meat along with other protein rich food groups estimated by the 24-hour dietary recall is presenting in **S17**. The average daily intake in the adult population for red and processed meat was 45.8 g and 22.4 g, respectively. The average daily intake of animal-source protein foods, the sum of red meat, poultry, eggs, and seafood was 132.3 g. Those in the lowest income group consumed less red meat, processed meat, animal-source protein foods, dairy, and poultry than the highest income group. However, they did consume more legumes and eggs than the highest income group. Red meat consumers consumed, on average, 101.2 g/day of red meat, 16.0 g/day of processed meat, and 162.4 g/day of animal-source protein foods. The average daily intake among processed meat consumers was 43.0 g of red meat, 70.6 g of processed meat, and 121.0 g of animal-source protein foods.

*Dietary Substitutions over time in Mexican Cohort*

The average age was 44.43 (SD 4.72) years. Most participants had at least a university degree (93.9%), over half (60.5%) were either overweight or obese, and a minority were of an indigenous background (4.8%).

We observed a sustained decline in both red and processed meat consumption over time among a majority of participants (53.9% and 55.1% of participants, respectively). Reported grams consumed per day of red, processed and total meat were statistically different between 2006 and 2008, 2008 and 2014, and 2006 and 2014 (p-value <0.01). Results for linear regressions may be found in **S20 Table**.

*Sensitivity Analysis*

*Meat by cut quality in ENIGH*

Overall, the weighted proportion of households that reported positive consumption of cheap cuts of meat was 30.3% and for more expensive cuts was 51.2%. There were statistical differences in positive consumption of both types of meat across income quintiles. In the lowest income quintile, 18.2% of households reported positive consumption of cheaper cuts of meat and 35.7% of more expensive cuts. In contrast, 36.9% and 59.6% of households from the highest income quintile reported positive consumption of cheaper and more expensive cuts of meat, respectively. In terms of budget shares, cheaper and more expensive cuts of meat represented 6.6% and 8.8% of total food expenditures overall. There were significant differences observed across income quintiles. In the highest income quintile, cheaper and more expensive meat represented a higher proportion of budget shares (6.5% and 11.7%, respectively) compared to the lowest income quintile (4.9% and 5.2%, respectively).
