# Supplementary material for: Taxes to red and processed meat to promote sustainable and healthy diets in Mexico
Source: PLoS One. 2025 Jun 27;20(6):e0326616. doi: 10.1371/journal.pone.0326616 (PMC12204545; doi:10.1371/journal.pone.0326616)
Supplement: S3 Text — (DOCX) [file pone.0326616.s004.docx]

# **S3 Text. Supplementary References**

[1] Secretaría de Salud (SSA), Instituto de Salud Pública (INSP, Alimentación y Medio Ambiente (GISAMAC). Guías alimentarias saludables y sostenibles para la población mexicana 2022 n.d.

[2] Willet W, Rockstrom J, Loken B, Springmann M. Food in the Anthropocene: the EAT–Lancet Commission on healthy diets from sustainable food systems - The Lancet 2019.

[3] Colchero MA, Salgado JC, Unar-Munguía M, Hernández-Ávila M, Rivera-Dommarco JA. Price elasticity of the demand for sugar sweetened beverages and soft drinks in Mexico. Econ Hum Biol 2015;19:129–37. https://doi.org/10.1016/j.ehb.2015.08.007.

[4] Moschini G. Units of Measurement and the Stone Index in Demand System Estimation. Am J Agric Econ 1995;77:63–8. https://doi.org/10.2307/1243889.

[5] Babu S, Gajanan S, Hallam JA. Nutrition Economics: Principles and Policy Applications. Academic Press; 2016.

[6] Lajous M, Ortiz-Panozo E, Monge A, Santoyo-Vistrain R, García-Anaya A, Yunes-Díaz E, et al. Cohort Profile: The Mexican Teachers’ Cohort (MTC). Int J Epidemiol 2017;46:e10. https://doi.org/10.1093/ije/dyv123.

[7] Hernández-Avila M, Romieu I, Parra S, Hernández-Avila J, Madrigal H, Willett W. Validity and reproducibility of a food frequency questionnaire to assess dietary intake of women living in Mexico City. Salud Publica Mex 1998;40:133–40. https://doi.org/10.1590/s0036-36341998000200005.

[8] USDA National Nutrient Database for Standard Reference, Legacy Release | Ag Data Commons n.d. https://data.nal.usda.gov/dataset/usda-national-nutrient-database-standard-reference-legacy-release (accessed May 23, 2023).
