# Supplementary material for: Taxes to red and processed meat to promote sustainable and healthy diets in Mexico
Source: PLoS One. 2025 Jun 27;20(6):e0326616. doi: 10.1371/journal.pone.0326616 (PMC12204545; doi:10.1371/journal.pone.0326616)
Supplement: Attachment — Submitted filename: Connors2024_Mexican_DG_Tax.docx [file pone.0326616.s005.docx]

**Promoting adherence to the sustainable Mexican Dietary Guidelines through a tax on red and processed meat**

Kaela Connors, MSc^1,2*^, Juan A. Rivera, PhD^3^, Peter Alexander, PhD^1,2^, Lindsay M. Jaacks, PhD^2^, Carolina Batis, PhD^4^, Dalia Stern, PhD^5^, Martín Lajous, ScD^3,6^, M. Arantxa Colchero, PhD^7^

^1^ School of Geosciences, University of Edinburgh, Edinburgh, UK

^2^ Global Academy of Agriculture and Food Systems, University of Edinburgh, Midlothian

^3^ Center for Research in Population Health, National Institute of Public Health, Cuernavaca, Morelos, Mexico

^4^ Center for Nutrition and Health Research, National Institute of Public Health, Cuernavaca, Morelos, Mexico

^5^ CONAHCyT-Center for Research on Population Health, National Institute of Public Health, Cuernavaca, Morelos, Mexico

^6^ Department of Global Health and Population, Harvard T.H. Chan School of Public Health, Boston, United States

^7^Center for Evaluation and Surveys, National Institute of Public Health, Cuernavaca, Morelos, Mexico

***Correspondence:**

Kaela Connors

School of Geosciences

Global Academy of Agriculture and Food Systems

University of Edinburgh

Edinburgh, UK

Telephone: +44 0131 650 6586

Email: [k.m.connors@sms.ed.ac.uk](mailto:k.m.connors@sms.ed.ac.uk)

**Abstract**

**Background**

Consumption of red and processed meat is above sustainable and healthy dietary targets in Mexico. Policies to promote greater adherence such as taxing meat are needed to reduce consumption. Here, we evaluated how price increases to red and processed meat could shift consumption for meat as well as other key food groups.

**Methods**

Using data from the Mexican National Household Income and Expenditure Survey (2018, 2020, 2022), we estimated own- and cross-price elasticities of demand for 10 food groups. These were used to predict changes in quantity demanded of each food group according to price increase scenarios.

**Results**

Price increases to meat increased demand for substitutes such as fruits and vegetables, legumes, poultry, and seafood, and reduced demand for salty snacks, sweets and sugary beverages. Substantial increases to the price of processed meat markedly reduced processed meat consumption, increased consumption of protein- and nutrient-rich foods, and resulted in more modest levels of red meat consumption. Lower-income groups were more sensitive to price increases but still met dietary recommendations for meat and substituted these foods with alternatives such as legumes and seafood.

**Conclusions**

Increasing the price of red and processed meat through a tax may promote greater adherence to sustainable and healthy dietary targets in Mexico. It simultaneously increased demand for healthier substitutes, and reduced demand for unhealthy complements. Substantially raising the price of processed meat only may be an effective strategy for addressing diet-related risk factors, while delivering environmental benefits. Additionally, meat taxes have the potential to promote improvements in diet quality and equitable health outcomes.

**Introduction**

Net zero emissions targets cannot be achieved without transforming food systems, which are responsible for one third of greenhouse gas emissions (1). Meat production accounts for a majority of these emissions (1). High consumption of red and processed meat is also associated with colorectal cancer (2) and type 2 diabetes (3), among other health risks (4,5). Global demand for meat is shifting from high- to middle-income countries and so more context-specific research is needed to inform pathways to sustainable and healthy diets in these geographies (6,7).

Adherence to sustainable and healthy diets remains low in Mexico (8,9) despite the integration of sustainability into the national Dietary Guidelines (10) and research suggesting that such diets are affordable in this context (11). In particular, meat consumption continues to increase and is well above recommended levels (8). Reducing red and processed meat consumption has been identified as key to increasing the sustainability of healthy diets in the Mexican context (8,9).

Government-supported demand-side interventions such as fiscal policies are one way to support food system transformations (12). Mexico was one of the first countries to pass a food-specific tax in 2014 on sugary beverages and an ad valorem tax on non-essential energy-dense food to address its diet-related chronic disease burden (13). Curbing demand for red meat may require more aggressive approaches such as higher rates of taxation (14,15). Hypothetical taxes on meat have previously been studied in Europe, the United States, and most recently, Brazil (14–18) but no study has evaluated such a tax in the Mexican context.

We assessed how to achieve sustainable, equitable, and healthy dietary targets for red and processed meat consumption in Mexico. The four specific aims were to (1) estimate own- and cross-price elasticities of red and processed meat, (2) estimate demand responses to price increases in red and processed meat under various scenarios and apply them to consumption data, (3) explore if price elasticities varied by household income and other effect modifiers (i.e., survey year, and consumption status) and (4) corroborate cross-sectional findings by evaluating dietary substitutions and complements to red and processed meat in a longitudinal cohort of Mexican women.

**Materials and Methods**

*Price elasticities for red and processed meat*

We used 2018, 2020, and 2022 data from the Mexican National Income and Expenditure Survey (ENIGH, by its Spanish acronym), administered by the Mexican National Institute of Statistics and Geography. The survey has a two-stage probabilistic design and collects cross-sectional nationally representative information on household income and expenditures including sociodemographic information every two years. It consolidates households’ daily food and beverage expenditures for one week, as reported by the head of household. After excluding households that did not report food expenditures or that only reported non-descript meal events outside the home, the unweighted analytical sample for all three years combined was 248,226 (**S1 Appendix**).

Food expenditures were categorized into 11 food groups: (1) dairy, (2) eggs, (3) salty snacks, sweets, and sugary beverages (herein, discretionary foods), (4) seafood, (5) fruits and vegetables (F&V) (6) legumes, nuts and seeds (herein, legumes), (7) grains, roots, and tubers (herein, grains), (8) unprocessed poultry and offal (herein, poultry), (9) processed meat (including processed red meat and poultry), (10) unprocessed red meat and offal (herein, red meat), and (11) other foods (i.e, spices, condiments, oils, non-sweetened beverages such as water, tea, and coffee)(**S1 Table**).

We calculated uncompensated own- and cross-price elasticities of demand for 10 food groups through estimation of a complete demand system using a linear approximate of an Almost Ideal Demand System (LA/AIDs)(19). The model was estimated using Ordinary Least Squares equation and ‘other foods’ was omitted to account for potential collinearity between food groups. Demand shifters included educational attainment of the head of household, year of survey (2018, 2020, and 2022), region of residence (urban vs rural), and adult equivalent based on estimations derived from the Mexican population (20). Price elasticities were first presented by pooling three survey rounds. We then calculated price elasticities stratified by: (1) survey round, (2) income quintile, (3) consumers of red meat (non-zero expenditures on red meat), and (4) consumers of processed meat (non-zero expenditures on processed meat). More details on model specifications are in **S1 Appendix**. We used the package for *Demand Analysis with the Almost Ideal Demand System* in R statistical software.

We tested price increases previously modeled in other settings and chose combinations where processed meat prices were higher than red meat due to more consistent evidence of processed meat’s adverse health effects (14,21)(**S1 Appendix; S1 Fig**).

Price Increase Scenarios:

1) Reference: no price increase
2) Scenario 1: price increase of 30% to both red and processed meat(16,17)
3) Scenario 2: 15% price increase to red meat and 30% to processed meat(16)
4) Scenario 3: 7% price increase for red meat and 47% to processed meat(14)
5) Scenario 4: 100% price increase to processed meat only(14)

We then obtained percent changes in quantity demanded of food groups included in the demand system by applying the price increase to meat and then calculating the change in demand of food groups using own- and cross-price elasticities. We performed the same procedure within each subpopulation.

In a sensitivity analysis, we separated meat into ‘expensive’ and ‘cheaper’ cut of meat according to price instead of red and processed meat given the heterogeneity of prices within the meat category and calculated price elasticities (**S1 Appendix; S2 Table**).

*Application to national consumption data*

Baseline consumption data were from a 24-hour dietary recall administered in 2016 by the Mexican National Health and Nutrition Survey (ENSANUT, by its Spanish acronym). ENSANUT uses a multi-stage probability design to sample the civilian, non-institutionalized population of Mexico. Data are obtained through face-to-face interviews performed in participants’ homes. The analytic sample for adults (≥ 18 years) who had completed the recall, were not pregnant or lactating, and had plausible energy values (total energy intake ≥400 kcal/day and ≤ 4,000 kcal/day) was 1,558 participants.

We categorized red and processed meat into their respective groups, including meat consumed in mixed dishes which were disaggregated using a recipe file developed by the National Institute of Public Health, Mexico (**S3 Table**). We also created ‘expensive’ and ‘cheaper’ cuts of meat categories using dietary consumption data from ENSANUT by matching items included in each category from the expenditure data when possible (**S4 Table**)

The estimated changes (%) in quantity demanded of red and processed meat which were translated to reductions in daily intake (g) for the Mexican adult population and subgroups (i.e., red meat consumers, processed meat consumers, lowest income, highest income) (**S1 Appendix**). Consumption after price increase was compared to dietary targets stipulated by the 2023 Mexican Dietary Guidelines (approximately 30g/day for red meat and 0g/day for processed meat) and the EAT-*Lancet* (approximately 14 g/day for red meat and 0 g/day for processed meat) based on an average adult consuming 2,500 kcal/day(10,22). We also calculated consumption of animal-source protein foods (the sum of red meat, poultry, eggs, and fish) before and after price increases to compare them to the Mexican and EAT-Lancet targets (140g/day and 84g/day, respectively **S1 Appendix**).

*Dietary substitutions using longitudinal dietary intake data*

To corroborate cross-sectional findings, we conducted a secondary analysis of a longitudinal cohort of Mexican women in the states of Jalisco and Veracruz. Data were from the Mexican Teachers’ Cohort (MTC), a prospective cohort study of 115,307 middle-aged female teachers across Mexico. MTC evaluated dietary intake using a validated 140-item food frequency questionnaire (FFQ). The sample size was 1,417 based on those who had completed the FFQ in all three waves where dietary data was collected (2006, 2008, and 2014) and based on additional eligibility criteria (**S1 Appendix**). We categorized food groups according to those modelled in the demand system (**S1 Appendix; S5 Table)**. Information on egg consumption was not collected in 2006 and therefore was not included.

We calculated changes in quantity consumed (g/day) for each period (Period 1: 2006-2008 and Period 2: 2008-2014). We then evaluated potential dietary substitution and complement behavior by applying a linear regression predicting changes in red, processed, and total meat intake with changes in non-meat food groups adjusting for changes in total daily energy intake (**S1 Appendix**).

**Results**

The own-price elasticities of red and processed meat in the pooled sample after adjustment were -0.79 and -0.83, respectively **(Table 1; S2 Appendix; Tables S6-S11)**. In other words, a 10% increase in the price of red meat would result in a 7·9% decrease in quantity demanded of red meat, and a 10% increase in the price of processed meat would result in an 8·3% decrease in quantity demanded of processed meat (**Table 1)**. Own-price elasticities for both red and processed meat decreased by survey year, meaning they became less sensitive to price (**S12 Table).** Stratified by income, the own-price elasticities of both red and processed meat decreased as income increased, meaning higher-income households were less sensitive to price increases in red and processed meat (**Fig.1**). In the lowest- and highest-income group, red meat was more elastic than processed meat, therefore, there were larger reductions in demand for red meat when price increased compared to processed meat for these groups. The own-price elasticity of red meat was more inelastic compared to processed meat in the middle-income quintiles.

**Table 1 – Own- and cross-price elasticity of demand for red and processed meat and other food groups**

| **Food Category** | **Own-price elasticity** | **Cross-price elasticity with red meat** | **Cross-price elasticity with processed meat** |
| --- | --- | --- | --- |
| **Dairy** | -1·54.(0.01)* | 0.08 (0.01)* | -0.11 (0.01)* |
| **Eggs** | -0.48 (0.04)* | 0.05 (0.03) | -0.20 (0.02)* |
| **Discretionary Foods** | -1.26 (0.00)* | -0.49 (0.01)* | -0.04 (0.00)* |
| **Seafood** | -2.09 (0.04)* | -0.41 (0.07)* | 0.34 (0.04)* |
| **Fruits and Vegetables,** | -1.77 (0.01)* | 0.42 (0.01)* | 0.01 (0.01) |
| **Grains, roots, and tubers** | -1.10 (0.01)* | 0.32 (0.01) | 0.02 (0.01)* |
| **Legumes, nuts, and seeds** | -0.95 (0.04)* | 0.07 (0.05)* | 0.37 (0.03)* |
| **Poultry** | -0.65 (0.01)* | -0.29 (0.02)* | 0.04 (0.01)* |
| **Processed Meat** | -0.83 (0.04)* | -0.09 (0.04)* | - |
| **Red Meat** | -0.79 (0.04)* | - | -0.04 (0.01)* |
| *Significant at the 1%. Non-compensated price elasticity standard errors in parentheses.  The ‘Other’ food group category was omitted to avoid collinearity in OLS estimation.  Demand shifters included educational attainment of the head of household, year of survey (2018, 2020, and 2022), region of residence (urban vs rural), adult equivalent based on estimations derived from the Mexican population. | | | |

**Figure 1 – Own-price elasticity of demand for red and processed meat by income quintile**

All estimations are significant at 1%.

The ‘Other’ food group category was omitted to avoid collinearity in OLS estimation.

Demand shifters included educational attainment of the head of household, year of survey (2018, 2020, and 2022), region of residence (urban vs rural), and adult equivalent based on estimations derived from the Mexican population.

An increase in the price of red meat was associated with an increase in the demand F&V, grains, dairy, and legumes indicating these food groups were substitutes to red meat (**Table 1**). In contrast, increases to red meat price were associated with decreases in demand for all other food groups including discretionary foods, meaning that these food groups were complements to red meat. Over time, seafood, legumes, and processed meat became stronger complements, F&V a stronger substitute, and dairy and eggs weaker substitutes to red meat (**S2** **Appendix, S13 Table**).

An increase in the price of processed meat was associated with an increase in demand for seafood, legumes, grains, and poultry. Complements for processed meat were eggs, dairy, red meat, and discretionary foods. Looking at the food groups with the highest cross-price elasticities, F&V was a strong substitute to red meat and legumes was a strong substitute to processed meat, whereas discretionary foods were a strong complement to red meat and eggs were a strong complement to processed meat (**Table 1**). Over time, F&V became a complement instead of a substitute, red meat became a stronger substitute, and poultry was no longer a substitute to processed meat (**S2 Appendix, S14 Table**).

Substitution and complement patterns varied by income group (**S15** **Table**). Comparing the highest to the lowest income group, discretionary foods, seafood, and processed meat were stronger complements to red meat. In the lowest income group, dairy, eggs, and F&V were stronger substitutes and poultry was a stronger complement to red meat. For processed meat, discretionary foods and red meat were stronger complements and poultry a stronger substitute in the highest income group compared to lowest. Moreover, F&V and seafood were stronger substitutes and dairy and eggs were stronger complements to processed meat in the lowest compared to highest income group.

*Price elasticities of meat by cut quality*

The own-price elasticities for cheaper and more expensive cuts of meat were -0.83 and -0.78, respectively (**S2 Appendix; Tables S16-S17; S2 Fig.**). Cheaper cuts of meat tended to be processed meat whereas more expensive cuts of meat tended to be red meat (hence, the similarity between own-price elasticities) (**Table S4)**. Therefore, we did not simulate demand responses to price increases by cut of meat.

*Applying tax scenarios*

Nearly half (45%) of Mexican adults consumed red meat and 32% consumed processed meat on a given day in 2016 (**S2 Appendix; S18 Table**).

When a price increase of 30% was applied to both red and processed meat (Scenario 1), red meat consumption decreased to levels near the Mexican Dietary Guidelines in the general adult population (34g/day vs. 30g/day recommended), but processed meat consumption did not (16g/day vs 0g/day recommended) (**Table 2**). Daily intake of animal-source protein foods increased from 132g/day to 145g/day vs. 140g/day and 84g/day recommended by the Mexican Dietary Guidelines and EAT-*Lancet*, respectively. The lowest income consumers came closer to red and processed meat dietary targets than the highest income consumers (**Table 2**). Red and processed meat consumers did not reduce demand for meat as much as the general adult population (**Table 2; Figs.2-3**). Scenario 1 led to the largest declines in percent demanded of discretionary foods, poultry, and red meat and the largest increases in demand for F&V and grains (**Fig.2**). For red meat and processed consumers, it resulted in the largest increases in F&V and grains and the largest decreases in discretionary foods, seafood, and red meat (**Fig.3**). Scenario 1 increased F&V consumption in the lowest income group and reduced discretionary foods in the highest income group (**Fig.4**).

After a 15% price increase for red meat and 30% for processed meat (Scenario 2), demand for red meat slightly decreased as red meat is a complement (**Table 2; Fig.2**). Scenario 3, which comprised of a 7% price increase to red meat and 47% price increase to processed meat, achieved levels of red and processed meat consumption aligned with the Mexican Dietary Guidelines in the lowest income group whereas among red meat consumers it resulted in minimal changes in demand of red meat (**Table 2; Figs.3-4**).

**Table 2 – Estimated percentage change in quantity demanded and average consumption (g/day) by population according to price increases to red and processed meat in Mexico**

|  | | **Red Meat** | | **Processed Meat** | | | | | **Animal-source protein foods** | |  |
| --- | --- | --- | --- | --- | --- | --- | --- | --- | --- | --- | --- |
|  | | **Reduction in quantity demanded, %** | **Consumption after price increase (g/day)** | **Reduction in quantity demanded, %** | | **Consumption after price increase (g/day)** | | **Consumption after price increase (g/day)** | | |  |
| **Reference: no price increase (baseline consumption)** | | | | | | | | |  | |  |
| **Adult Population ≥18 years of age** | | 0 | 45.8 (4.9) | 0 | | 22.4 (3.1) | | 132.3 (6.9) | | |  |
| **Consumption Status** | |  |  |  | |  | |  | | |  |
| Red Meat Consumers | | 0 | 101.2 (9.6) | 0 | | 16.0 (2.6) | | 162.4 (12.8) | | |  |
| Processed Meat Consumers | | 0 | 43.0 (11.8) | 0 | | 70.6 (7.2) | | 121.0 (11.0) | | |  |
| **Income groups** | |  |  |  | |  | |  | | |  |
| Lowest | | 0 | 26.3 (5.4) | 0 | | 11.2 (4.5) | | 100.2 (12.6) | | |  |
| Highest | | 0 | 56.2 (9.7) | 0 | | 28.2 (5.9) | | 144.1 (12.4) | | |  |
| **Scenario 1 – 30% price increase both red and processed meat** | | | | | | | | |  | |  |
| **Adult Population ≥18 years of age** | | -24.7% | 34.5 | -27.8% | | 16.2 | | 144.9 | | |  |
| **Consumption Status** | |  |  |  | |  | |  | | |  |
| Red Meat Consumers | | -19.5% | 81.4 | -27.8% | | 11.6 | | 161.5 | | |  |
| Processed Meat Consumers | | -25.4% | 32.1 | -19.2% | | 57.1 | | 130.8 | | |  |
| **Income groups** | |  |  |  | |  | |  | | |  |
| Lowest | | -35.4% | 17.0 | -28.9% | | 8.0 | | 132.4 | | |  |
| Highest | | -18.7% | 45.7 | -22.8% | | 21.8 | | 153.8 | | |  |
| **Scenario 2– 15% price increase red meat and 30% price increase processed meat** | | | | | | | | |  | |  |
| **Adult Population ≥18 years of age** | | -12·9% | 39.9 | -26.4% | | 16.4 | | 152.3 | | |  |
| **Consumption Status** | |  |  |  | |  | |  | | |  |
| Red Meat Consumers | | -9·8% | 91.3 | -26.8% | | 11.7 | | 174.2 | | |  |
| Processed Meat Consumers | | -17·5% | 36.7 | -15.4% | | 58.2 | | 136.7 | | |  |
| **Income quintiles** | |  |  |  | |  | |  | | |  |
| Lowest | | -18·3% | 21.5 | -27.7% | | 8.1 | | 138.4 | | |  |
| Highest | | -10·5% | 50.3 | -19.1% | | 22.8 | | 160.7 | | |  |
| **Scenario 3 –7% price increase red meat and 47% price increase processed meat** | | | | | | | | |  | |  |
| **Adult Population ≥18 years of age** | | -7.3% | 42.5 | -39.9% | | 13.5 | | 157.4 | | |  |
| **Consumption Status** | |  |  |  | |  | |  | | |  |
| Red Meat Consumers | | -4.5% | 96.6 | -40.9% | | 9.5 | | 182.1 | | |  |
| Processed Meat Consumers | | -13·1% | 37.4 | -25.6% | | 52.5 | | 139.7 | | |  |
| **Income groups** | |  |  |  | |  | |  | | |  |
| Lowest | | -9·8% | 23.7 | -42.0% | | 6.7 | | 142.0 | | |  |
| Highest | | -7·5% | 52.0 | -25.8% | | 21.0 | | 165.1 | | |  |
| Values in reference scenario are survey-weighted mean (SE) grams per day consumer per capita. Animal-source protein foods refers to the sum of red meat, fish, poultry, and eggs consumed per day. Animal-source protein food daily intake was determined as the sum of red meat, poultry, eggs, and fish as describe in **Table S1.**  The average grams/day were calculated applying the percentage reduction in demand to actual single-day weighted intake for each population. For animal-source protein foods, the percent changes in demand were applied to baseline consumption of each respective food group and then summed to obtain estimated daily intake after price increase. | | | | | | | | | | |  |
|  | **Red Meat** | | | | **Processed Meat** | | | | | **Animal-source protein foods** | |
|  | **Reduction in quantity demanded, %** | | **Consumption after price increase (g/day)** | | **Reduction in quantity demanded, %** | | **Consumption after price increase (g/day)** | | | **Consumption after price increase (g/day)** | |
| **Scenario 4 – 100 % price increase to processed meat only** | | | | | | | | | |  | |
| **Adult Population ≥18 years of age** | -3.8% | | 44.0 | | -83.5% | | 3.7 | | | 164.4 | |
| **Consumption Status** |  | |  | |  | |  | | |  | |
| Red Meat Consumers | -0.05% | | 101.2 | | -86.0% | | 2.2 | | | 191.7 | |
| Processed Meat Consumers | -18.1% | | 35.2 | | -52.8% | | 33.4 | | | 141.7 | |
| **Income groups** |  | |  | |  | |  | | |  | |
| Lowest | -3.7% | | 25.3 | | -88.3% | | 1.3 | | | 146.0 | |
| Highest | -7.9% | | 51.7 | | -51.2% | | 13.8 | | | 170.8 | |
| Values in reference scenario are survey-weighted mean (SE) grams per day consumer per capita. Animal-source protein foods refers to the sum of red meat, fish, poultry, and eggs consumed per day. Animal-source protein food daily intake was determined as the sum of red meat, poultry, eggs, and fish as describe in **Table S1.**  The average grams/day were calculated applying the percentage reduction in demand to actual single-day weighted intake for each population. For animal-source protein foods, the percent changes in demand were applied to baseline consumption of each respective food group and then summed to obtain estimated daily intake after price increase. | | | | | | | | | | | |

**Figure 2– Estimated percent change in quantity demanded according to price increases in red meat and processed in Mexican Adults**

Values are percentage change in quantity demanded of food groups according to four price increase scenarios for red and processed meat estimated by own- and cross-price elasticities. **Scenario 1**: 30% price increase to both red and processed meat; **Scenario 2:** a 15% price increase to red meat and a 30% price increase to processed meat; **Scenario 3:** a 7% price increase to red meat and a 47% price increase to processed meat; **Scenario 4:** 100% price increase to processed meat only.

F&V refers to ‘fruits and vegetables’, Grains refers to ‘Grains, roots, and tubers’, Legumes refers to ‘legumes, nuts, and seeds’, and Proc Meat refers to processed meat.

**Figure 3 – Estimated percent change in quantity demanded according to price increases in red meat and processed among consumers**

**Top graph** Values are percentage change in quantity demanded of food groups for red meat consumers according to four price increase scenarios for red and processed meat based on own- and cross-price elasticities calculated for households who reported non-zero red meat consumption. **Scenario 1**: 30% price increase to both red and processed meat; **Scenario 2:** a 15% price increase to red meat and a 30% price increase to processed meat; **Scenario 3:** a 7% price increase to red meat and a 47% price increase to processed meat; **Scenario 4:** 100% price increase to processed meat.

**Bottom graph**: Values are percentage change in quantity demanded of food groups for processed meat consumers according to four price increase scenarios for red and processed meat based on own- and cross-price elasticities calculated for households who reported non-zero processed meat consumption. **Scenario 1**: 30% price increase to both red and processed meat; **Scenario 2:** a 15% price increase to red meat and a 30% price increase to processed meat; **Scenario 3:** a 7% price increase to red meat and a 47% price increase to processed meat; **Scenario 4:** 100% price increase to processed meat only.

F&V refers to ‘fruits and vegetables’, Grains refers to ‘Grains, roots, and tubers’, Legumes refers to ‘legumes, nuts, and seeds’, and Proc Meat refers to processed meat

**Figure 4 – Estimated percent change in quantity demanded according to price increases in red meat and processed among low- and high-income groups**

**Top graph** Values are percentage change in quantity demanded of food groups for low-income consumers according to four price increase scenarios for red and processed meat based on own- and cross-price elasticities calculated for households in lowest income quintile. **Scenario 1**: 30% price increase to both red and processed meat; **Scenario 2:** a 15% price increase to red meat and a 30% price increase to processed meat; **Scenario 3:** a 7% price increase to red meat and a 47% price increase to processed meat; **Scenario 4:** 100% price increase to processed meat only.

**Bottom graph**: Values are percentage change in quantity demanded of food groups for high income consumers according to three price increase scenarios for red and processed meat based on own- and cross-price elasticities calculated for households in highest income quintile. **Scenario 1**: 30% price increase to both red and processed meat; **Scenario 2:** a 15% price increase to red meat and a 30% price increase to processed meat; **Scenario 3:** a 7% price increase to red meat and a 47% price increase to processed meat; **Scenario 4:** 100% price increase to processed meat only.

F&V refers to ‘fruits and vegetables’, Grains refers to ‘Grains, roots, and tubers’, Legumes refers to ‘legumes, nuts, and seeds’, and Proc Meat refers to processed meat.

A 100% price increase to processed meat only (Scenario 4) aligned red and processed consumption closely to the Mexican Dietary Guidelines resulting in the largest declines in processed meat across all subgroups compared to other scenarios (**Table 2; Figs.2-4**). For the general adult, red meat consumer, and lowest income population, demand for processed meat reduced by approximately 84% with slight reductions in red meat due to their complementarity. This scenario resulted in the greatest reductions in processed meat among processed meat consumers and moderately reduced demand for red meat since red meat is a stronger complement to processed meat in this group compared to the reverse in red meat consumers (**Fig.4**). Scenario 4 led to the largest declines in dairy and eggs but largest increases in seafood, legumes, and poultry in the adult population (**Fig.2**). It also achieved the greatest consumption of animal-source protein foods in the lowest income group (**Table 2**). For red meat consumers, it led to the largest increases in seafood and legumes whereas among processed meat consumers it led to the largest increases in legumes and eggs (**Fig.3**). In the lowest income group, it resulted in greater increases in demand for seafood and F&V whereas in the highest income group it led to greater increases in demand for legumes and poultry (**Fig.4**).

Lastly, since no predefined price increase scenarios reached the processed meat 0g/day target, we calculated that an approximate 120% price increase would be required for processed meat to achieve such a target in the adult Mexican population.

*Dietary substitutions over time in MTC*

Red and processed meat were also complements to each other in a longitudinal study of Mexican women (**S19 Table**). Changes in red, processed, and total meat intake from 2006-2008 and 2008-2014 were negatively correlated with changes in F&V, supporting evidence that this food group is a substitute for red and total meat (**S19 Table**). Changes in processed and total meat intake were positively correlated with changes in poultry, supporting this food group as a complement. Moreover, changes in seafood intake were positively correlated with processed and total meat intake in both periods suggesting this food group is a complement. Lastly, we did not find correlations between changes of red, processed, or total meat intake with discretionary foods, dairy, nor grains.

**Discussion**

Moderate price increases to meat in Mexico could help the general adult population achieve the Mexican Dietary Guidelines’ target for red meat, but more substantial increases in the price of processed meat (about 120%) are required to achieve the target of 0g/day. The own-price elasticities for red and processed meat are relatively similar; therefore, increasing the price by the same ratio results in similar reductions in demand for each. However, it results in different changes in demand for other food groups important for nutrition and health (10). As red meat consumption goes down, consumption of F&V, grains, and dairy is likely to go up and consumption of discretionary foods, poultry, seafood, and processed meat is likely to go down. As processed meat consumption goes down, legumes, seafood, poultry, and grains are likely to go up and consumption of dairy, eggs, discretionary foods, and red meat is likely to go down. Increases in the price of red and processed meat increased consumption of nutrient- and protein-rich foods (legumes, seafood, and poultry) in the lowest income group. The Mexican Dietary Guidelines recommend replacing red and processed meat with beans, lentils, egg, poultry, and fish (10). Therefore, increasing the price of red and processed meat may promote greater adherence to the guidelines especially in lower income groups.

We found that a substantial price increase for processed meat alone (i.e., a 100% increase) may be a better strategy than moderately increasing the price of both red and processed meat. Processed meat poses greater health burden (23) and has been consistently found to require higher pricing than red meat to internalize health costs (14). This scenario presented the highest increases in protein-rich foods such as seafood, legumes, and poultry out of the four scenarios in all groups and also increased F&V. It maintained consumption of red meat at recommended levels in the lowest income group, for whom moderate consumption of may be important to ensure sufficient intake of key nutrients (8,24).

Our findings are in agreement within the context of the relevant literature. LA/AIDs was previously carried out using ENIGH 2002-2012 in Mexico for urban households only and derived an own-price elasticity of approximately -0.803 for meats (including red, processed meats, and poultry) (25). In this study, the own-price elasticity for seafood was also the highest among the food groups as it was in our findings further corroborated in another study comparing pre- and during COVID-19 food demand in Mexico (-2.07 for seafood before COVID-19) (26). A meta-analysis of global own-price elasticities for meat from 2010 obtained a median own-price elasticity for meat (including ruminant, non-ruminant, poultry, and fish) of -0.762 (27). More specifically, a pooled own-price elasticity of -0.87 was obtained for beef and -0.65 for poultry (27). Our own-price elasticities are roughly consistent with these estimates despite difficulty in comparison due to heterogeneity in food groupings and context-specific demand systems. We found that red and processed meat became more inelastic over time (especially after 2020) and hypothesize this might be due to stronger complements (such as seafood, legumes and F&V) and weaker substitutes (such as dairy, eggs, and poultry) to red and processed meat over time.

Previous studies have proposed taxes on meat to internalize negative externalities associated with high meat consumption, be it environmental consequences, human health, animal welfare, or social consequences including labor (28). In Sweden, France, and the Netherlands, price increases ranging between 15% to 34% were modeled for beef, all animal products, and total meat (including red, white, and processed) (16,17,29) and in Brazil these ranged from 5% to 60% for beef to account for environmental and health costs (18). A global modeling study found that the price of red meat should increase on average by 21.26% for high-income and 6.51% for upper-middle income countries to account for associated health costs (14). Processed meat required greater price increases due to its greater health burden: 111% for high-income countries (up to 200% in the United States) and 46·85% for upper middle-income countries (14). In the real world, meat taxes have mostly been considered in Europe, but they are also being implemented and discussed in Latin America (18,30,31). For example, Colombia recently passed a tax on ultra-processed food that includes processed meats, ‘*embutidos*’(30). Brazil is currently proposing a health- and sustainability-motivated tax to ultra-processed foods that includes some processed meats(31).

We found that lower income groups were more responsive to changes in prices of red and processed meat. Moreover, in this case, lower income groups consumed less red and processed meat and spent a lower proportion of their food budget on these foods. In the case of health-motivated taxes, reductions in consumption among lower income groups can actually mitigate health disparities and healthcare expenditures (32). In addition, tax revenue recycling in the form of targeted lump sum transfers can be used to benefit lower income groups and counteract any potential regressivity of the tax (33). Furthermore, they can be used to subsidize the cost of healthier, more nutrient-rich foods such as F&V and legumes especially since both food groups were stronger substitutes to red and processed meat in the lowest compared to highest income group. This was explored in a recent study evaluating the regressivity of meat taxes across Europe (33). It found that tax recycling through lump sum transfers to the lowest quintile were more equitable than lowering the price of fruits and vegetables (33). Moreover, a social-cost benefit analysis applied price elasticities obtained from a literature review to test price increases of 15 and 30% to total meat (including red, white and processed) and found that a 10% decrease in price of fruit and vegetables counteracted regressivity among lower income groups (16).

This analysis has many strengths in that it estimated the Mexican food demand system pooling three survey rounds of nationally representative household expenditure data to estimate price elasticities of key food groups. Only two previous studies (17,29) that modeled meat taxes calculated price elasticities based on the country-specific food demand systems as the other papers either used price elasticities from literature reviews (16) or calculated the marginal costs of consumption to health (14). Additionally, we were able to evaluate demand responses to price in the Mexican population as well as by income, and apply these scenarios to actual red and processed meat intake based on dietary data. Lastly, we further confirmed these findings in an independent analysis of longitudinal dietary behaviors.

However, these findings should be interpreted with consideration of their limitations. First, price elasticities were modeled using LA/AIDs meaning that the relationship between price and demand was assumed to be linear. Future work may seek to determine whether there is non-linearity in the relationship meaning price increases would result in non-linear responses in demand. Second, we excluded food expenditures for consumption away from the home given since information on specific foods purchased was not collected in ENIGH (comprising approximately 16% of household food budgets across all three ENIGH survey rounds) especially among higher income households(34) which may have introduced bias. Third, we acknowledge that we cannot rule out potential bias in the model that may have influenced the relationship between price and demand. But we were able to account for multiple potential sources of endogeneity such as price outliers and demand shifters that may influence the relationship between price and quantity demanded in addition to calculating unit values at the municipality level to reduce this risk. Fourth, it should be noted that we are only modeling demand responses to price increases and not supplier responses. It cannot be assumed that declines in consumption will result in declines in production as Mexican livestock producers may choose to export instead of sell domestically which may result in ‘carbon leakage’(35). Nonetheless, beef is an international food commodity so no matter where it is produced, reducing its consumption may mitigate its contribution to environmental change.

Future studies should examine the potential to explore combined policy mechanisms such as front-of-package labeling or signaling (such as earmarking or public framing) in combination with taxes given combined policy mechanisms have been found to be more effective in trial settings since price is not the only determinant of food choice (15,36,37). Moreover, better understanding of the nutritional implications of such reductions to ensure that substitution patterns adequately deliver energy and nutrient requirements is needed. Additionally, a political analysis examining the key stakeholders and political instruments that would facilitate the passing of such legislation – as was done for the 2014 tax in Mexico (38) – would be beneficial considering the large reductions in demand required to meet sustainable and healthy dietary targets. Lastly, research should estimate the impact of these taxes on environment outcomes.

This study provides evidence required for the design of a tax which may be used to inform policymakers in Mexico. Increasing the price of red and processed meat in Mexico may be effective in reducing consumption to address both health- and environment- related challenges. Specifically, substantially increasing the price of processed meat may be the best option for maximum health gains while reducing environmental costs. Lower income groups are more sensitive to price increases in red and processed meat, but there is evidence they are substituting these foods with other protein- and nutrient-rich foods such as seafood, poultry, and legumes. Moreover, a tax may be combined with tax recycling or lump sum transfers to make these options more affordable to lower income groups. Government-supported price increases may be an effective way to shifting consumption to more sustainable and healthy diets.

**Author contributions**

**Conceptualization:** Kaela Connors, M. Arantxa Colchero, Juan A. Rivera, Peter Alexander, Lindsay M. Jaacks

**Data Curation:** Carolina Batis, Dalia Stern, Martín Lajous.

**Formal Analysis:** Kaela Connors with guidance from M. Arantxa Colchero.

**Writing – original draft:** Kaela Connors.

**Writing – review & editing:** Kaela Connors, Juan A. Rivera, Peter Alexander, Lindsay M. Jaacks, Carolina Batis, Dalia Stern, Martín Lajous, M. Arantxa Colchero.

**Acknowledgements**

We would like to thank Arne Heningsen, author of the micEconAids package in R, for guidance on its implementation and answering our queries. Kaela Connors received financial support from the Edinburgh Earth, Ecology, and Environment Doctoral Training Partnership (E4 DTP).

**Data Availability**

The household income and expenditure data are available through the Mexican National Institute of Statistics and Geography (INEGI) [https://en.www.inegi.org.mx/programas/enigh/](about:blank). The national diet data are available through the National Health and Nutrition Survey (ENSANUT) [https://ensanut.insp.mx/](about:blank). Data for the longitudinal cohort of middle-aged Mexican women (Mexican Teacher’s Cohort) are available upon request to [esmaestras@insp.mx](about:blank).

**References**

1.	Clark MA, Domingo NGG, Colgan K, Thakrar SK, Tilman D, Lynch J, et al. Global food system emissions could preclude achieving the 1.5° and 2°C climate change targets. Science. 2020 Nov 6;370(6517):705–8.

2.	Bouvard V, Loomis D, Guyton KZ, Grosse Y, Ghissassi FE, Benbrahim-Tallaa L, et al. Carcinogenicity of consumption of red and processed meat. Lancet Oncol. 2015 Dec;16(16):1599–600.

3.	Gu X, Drouin-Chartier JP, Sacks FM, Hu FB, Rosner B, Willett WC. Red meat intake and risk of type 2 diabetes in a prospective cohort study of United States females and males. Am J Clin Nutr. 2023 Dec;118(6):1153–63.

4.	Rouhani MH, Salehi-Abargouei A, Surkan PJ, Azadbakht L. Is there a relationship between red or processed meat intake and obesity? A systematic review and meta-analysis of observational studies: Red or processed meat and obesity. Obes Rev. 2014 Sep;15(9):740–8.

5.	Abete I, Romaguera D, Vieira AR, Munain AL de, Norat T. Association between total, processed, red and white meat consumption and all-cause, CVD and IHD mortality: a meta-analysis of cohort studies. Br J Nutr. 2014 Sep;112(5):762–75.

6.	Zeng L, Ruan M, Liu J, Wilde P, Naumova EN, Mozaffarian D, et al. Trends in Processed Meat, Unprocessed Red Meat, Poultry, and Fish Consumption in the United States, 1999-2016. J Acad Nutr Diet. 2019 Jul 1;119(7):1085-1098.e12.

7.	Stewart C, Piernas C, Cook B, Jebb SA. Trends in UK meat consumption: analysis of data from years 1–11 (2008–09 to 2018–19) of the National Diet and Nutrition Survey rolling programme. Lancet Planet Health. 2021 Oct 1;5(10):e699–708.

8.	Castellanos-Gutiérrez A, Sánchez-Pimienta TG, Batis C, Willett W, Rivera JA. Toward a healthy and sustainable diet in Mexico: where are we and how can we move forward? Am J Clin Nutr. 2021 May 1;113(5):1177–84.

9.	Curi-Quinto K, Unar-Munguía M, Rodríguez-Ramírez S, Rivera JA, Fanzo J, Willett W, et al. Sustainability of Diets in Mexico: Diet Quality, Environmental Footprint, Diet Cost, and Sociodemographic Factors. Front Nutr. 2022 May 27;9:855793.

10.	Secretaría de Salud (SSA), Instituto de Salud Pública (INSP, Alimentación y Medio Ambiente (GISAMAC). Guías alimentarias saludables y sostenibles para la población mexicana 2022. 2022;

11.	Batis C, Marrón-Ponce JA, Stern D, Vandevijvere S, Barquera S, Rivera JA. Adoption of healthy and sustainable diets in Mexico does not imply higher expenditure on food. Nat Food. 2021 Oct;2(10):792–801.

12.	Rivera JA, Colchero MA, Pérez-Ferrer C, Barquera S. Perspective: Mexico’s Experience in Building a Toolkit for Obesity and Noncommunicable Diseases Prevention. Adv Nutr. 2024 Mar 1;15(3):100180.

13.	Batis C, Rivera JA, Popkin BM, Taillie LS. First-Year Evaluation of Mexico’s Tax on Nonessential Energy-Dense Foods: An Observational Study. Wareham NJ, editor. PLOS Med. 2016 Jul 5;13(7):e1002057.

14.	Springmann M, Mason-D’Croz D, Robinson S, Wiebe K, Godfray HCJ, Rayner M, et al. Health-motivated taxes on red and processed meat: A modelling study on optimal tax levels and associated health impacts. PloS One. 2018;13(11):e0204139.

15.	Taillie LS, Bercholz M, Prestemon CE, Higgins ICA, Grummon AH, Hall MG, et al. Impact of taxes and warning labels on red meat purchases among US consumers: A randomized controlled trial. PLOS Med. 2023 Sep 18;20(9):e1004284.

16.	Broeks MJ, Biesbroek S, Over EAB, van Gils PF, Toxopeus I, Beukers MH, et al. A social cost-benefit analysis of meat taxation and a fruit and vegetables subsidy for a healthy and sustainable food consumption in the Netherlands. BMC Public Health. 2020 May 11;20(1):643.

17.	Säll S, Gren IM. Effects of an environmental tax on meat and dairy consumption in Sweden. Food Policy. 2015 Aug;55:41–53.

18.	Tavares M, Cunha BSL, Cruz T, Morais TN, Portugal-Pereira J. The influence of pricing interventions in food choices on Brazil: An agent-based modelling approach. Sustain Prod Consum. 2024 Jan 1;44:250–62.

19.	Deaton A, Muellbauer J. An Almost Ideal Demand System. Am Econ Rev. 1980;70(3):312–26.

20.	Teruel G, Rubalcava L, Santana A. ESCALAS DE EQUIVALENCIA PARA MÉXICO.

21.	Lescinsky H, Afshin A, Ashbaugh C, Bisignano C, Brauer M, Ferrara G, et al. Health effects associated with consumption of unprocessed red meat: a Burden of Proof study. Nat Med. 2022 Oct;28(10):2075–82.

22.	Willet W, Rockstrom J, Loken B, Springmann M. Food in the Anthropocene: the EAT–Lancet Commission on healthy diets from sustainable food systems - The Lancet. 2019.

23.	International Agency for Research on Cancer. IARC Monographs on the Evaluation of Carcinogenic Risks to Humans: Red and Processed Meat. 2018;114.

24.	ENSANUT. Resultados Nacionales, 2018-2019. 2019.

25.	Juarez-Torres M, editor. The Impact of Food Price Shocks on Consumption and Nutritional Patterns of Urban Mexican Households. 2015. 43 p.

26.	Vargas-Lopez A, Cicatiello C, Principato L, Secondi L. Consumer expenditure, elasticity and value of food waste: A Quadratic Almost Ideal Demand System for evaluating changes in Mexico during COVID-19. Socioecon Plann Sci. 2022 Aug 1;82:101065.

27.	Gallet CA. Meat Meets Meta: A Quantitative Review of the Price Elasticity of Meat. Am J Agric Econ. 2010;92(1):258–72.

28.	Funke F, Mattauch L, Van Den Bijgaart I, Godfray C, Hepburn CJ, Klenert D, et al. Is Meat Too Cheap? Towards Optimal Meat Taxation. SSRN Electron J [Internet]. 2021 [cited 2023 Dec 12]; Available from: https://www.ssrn.com/abstract=3801702

29.	Caillavet F, Fadhuile A, Nichèle V. Taxing animal-based foods for sustainability: environmental, nutritional and social perspectives in France. Eur Rev Agric Econ. 2016 Sep 1;43(4):537–60.

30.	Departamento Administrativo de la Funcion Publica. Ley 2277 de 2022. Congreso de Colombia. 2022;

31.	ACT P de S. Proposals to fight hunger, promote health, and protect the environment in the context of the brazilian Tax Reform - Posts | ACT BR [Internet]. 2024 [cited 2024 Jun 24]. Available from: https://actbr.org.br/post/proposals-to-fight-hunger-promote-health-and-protect-the-environment-in-the-context-of-the-brazilian-tax-reform/19721/

32.	Mytton OT, Clarke D, Rayner M. Taxing unhealthy food and drinks to improve health. BMJ. 2012 May 15;344:e2931.

33.	Klenert D, Funke F, Cai M. Meat taxes in Europe can be designed to avoid overburdening low-income consumers. Nat Food. 2023 Oct;4(10):894–901.

34.	A Langellier B. Consumption and expenditure on food prepared away from home among Mexican adults in 2006. Salud Pública México. 2015 Jan 7;57(1):4.

35.	Abadie LM, Galarraga I, Milford AB, Gustavsen GW. Using food taxes and subsidies to achieve emission reduction targets in Norway. J Clean Prod. 2016 Oct 15;134:280–97.

36.	Faccioli M, Law C, Caine CA, Berger N, Yan X, Weninger F, et al. Combined carbon and health taxes outperform single-purpose information or fiscal measures in designing sustainable food policies. Nat Food. 2022 May;3(5):331–40.

37.	Colchero MA, Paraje G, Popkin BM. The impacts on food purchases and tax revenues of a tax based on Chile’s nutrient profiling model. PLOS ONE. 2021 Dec 2;16(12):e0260693.

38.	James E, Lajous M, Reich MR. The Politics of Taxes for Health: An Analysis of the Passage of the Sugar-Sweetened Beverage Tax in Mexico. Health Syst Reform. 2020 Dec 1;6(1):e1669122.

**Supplementary Information**

# **S1 Appendix. Supplementary Methods**

## **Table S1. Categorization of Food Expenditures by Food Group in Mexican National Household Income and Expenditure Survey 2018, 2020, and 2022**

# **S1 Figure. Price increase ratios (%) for red and processed meat to achieve Mexican Dietary Guideline 2023 and EAT-Lancet Dietary Targets for Adult Mexican Population**

Based on own-and cross-price elasticities for red and processed meat assuming linearity in relationship between price and demand for red and processed meat.

Price increase ratios represent those that would be required to achieve Mexican DG 2023 Targets for red and processed meat (30g/day and 0g/day, respectively) and to achieve EAT-Lancet Dietary Targets for red and processed meat (14g/day and 0g/day, respectively).

A percent reduction of 34% and 100% for red and processed meat was calculated for the Mexican adult population based on current consumption levels to achieve Mexican DG 2023 targets.

A percent reduction of 69.2% and 100% for red and processed meat was calculated for the Mexican adult population based on current consumption levels to achieve EAT-Lancet Dietary Targets.

# **S2 Table. Categorization of Cuts of Red and Processed Meat in the Mexican National Household Income and Expenditure Survey 2018, 2020, and 2022**

# **S3 Table S3. Categorization of Red and Processed Meat in the Mexican National Health and Nutrition Survey (ENSANUT 2016)**

# **S4 Table. Categorization of Cuts of Meat in the Mexican National Health and Nutrition Survey (ENSANUT 2016)**

Includes both red and processed meat. Items were grouped to reflect categorization of ENIGH meat expenditures determined by price.

For ENIGH categorization, cheaper cuts of meat were defined as individual items with a price per unit ≤ 114.00 MXN (the weighted national average price per unit across all three survey rounds for red and processed meat combined). Expensive cuts of meat were defined as individual items with a price per unit > 114.00 MXN.

# **S5 Table. Categorization of Food Groups in the Mexican Teacher’s Cohort**

Items were grouped to reflect categorizations in ENIGH food items.

*Mixed dishes were disaggregated into their respective ingredient components based on a Mexican Teacher’s Cohort recipe file.

# **S6 Table. Sociodemographic characteristics of ENIGH households in Mexico, by survey year and for total sample, 2018-2022**

Mean (SE). Other values are percentages as indicated.

Household income is reported as mean (SE) and in Mexican pesos (MXN).

Statistics are weighted taking into account expansion factors and complex survey design.

Sample sizes are unweighted.

# **S7 Table. Proportion of Households with Non-Zero Consumption per Food Group in ENIGH by Survey Round and in Total Sample**

Weighted percentages.

*Chi-squared test with Rao & Scott’s second-order correction. Bolded p-values are significant at the alpha = 0.05 level of significance.

# **S8 Table- Proportion of Households with Non-Zero Consumption per Food Group in ENIGH by Income Quintile**

Weighted percentages.

*Chi-squared test with Rao & Scott’s second-order correction. Bolded p-values are significant at the alpha = 0.05 level of significance.

# **S9 Table. Average per unit value per Food Group in ENIGH by Survey Round and in Total Sample**

Weighted average (SE). Unit values were derived by dividing food group expenditure by units purchased. Per unit values are reported in Mexican pesos (MXN).

*Wilcoxon rank-sum test for complex survey samples. Bolded p-values are significant at the alpha = 0.05 level of significance.

# **S10 Table. Budget Share of Total Food Expenditure per Food Group in ENIGH by Survey Round and in Total Sample**

Weighted average share, % (SE). Budget shares were derived by dividing expenditures in food group by total food expenditures.

*Wilcoxon rank-sum test for complex survey samples. Bolded p-values are significant at the alpha = 0.05 level of significance.

# **S11 Table. Budget Share of Total Food Expenditure per Food Group in ENIGH by Income Quintile**

Weighted average share, % (SE). Budget shares were derived by dividing expenditures in food group by total food expenditures.

*Wilcoxon rank-sum test for complex survey samples. Bolded p-values are significant at the alpha = 0.05 level of significance.

# **S12 Table. Own-price elasticity of demand for red meat and other food groups by year**

*Significant at the 1%. Non-compensated price elasticity standard errors in parentheses.

The ‘Other’ food group category was omitted to avoid collinearity in OLS estimation.

Demand shifters included educational attainment of the head of household, region of residence (urban vs rural), adult equivalent based on estimations derived from the Mexican population.

# **S13 Table. Cross-price elasticity of demand for red meat and other food groups by year**

*Significant at the 1%. Non-compensated price elasticity standard errors in parentheses.

The ‘Other’ food group category was omitted to avoid collinearity in OLS estimation.

Demand shifters included educational attainment of the head of household, region of residence (urban vs rural), and adult equivalent based on estimations derived from the Mexican population.

# **S14 Table. Cross-price elasticity of demand for processed meat and other food groups by year**

*Significant at the 1%. Non-compensated price elasticity standard errors in parentheses.

The ‘Other’ food group category was omitted to avoid collinearity in OLS estimation.

Demand shifters included educational attainment of the head of household, region of residence (urban vs rural), and adult equivalent based on estimations derived from the Mexican population.

# **S15 Table. Own- and cross-price elasticity of demand for red meat and other food groups comparing lowest to highest income quintile**

*Significant at the 1%. Non-compensated price elasticity standard errors in parentheses.

The ‘Other’ food group category was omitted to avoid collinearity in OLS estimation.

Demand shifters included educational attainment of the head of household, year of survey (2018, 2020, and 2022), region of residence (urban vs rural), adult equivalent based on estimations derived from the Mexican population.

# **S16 Table. Own- and cross-price elasticity of demand for meat and other food groups**

*Significant at the 1%. Non-compensated price elasticity standard errors in parentheses.

The ‘Other’ food group category was omitted to avoid collinearity in OLS estimation.

Demand shifters included educational attainment of the head of household, year of survey (2018, 2020, and 2022), region of residence (urban vs rural), adult equivalent based on estimations derived from the Mexican population.

# **S2 Figure. Own-price elasticity of demand for expensive and cheap cuts of meat by income quintile**

All estimations are significant at 1%.

The ‘Other’ food group category was omitted to avoid collinearity in OLS estimation.

Demand shifters included educational attainment of the head of household, year of survey (2018, 2020, and 2022), region of residence (urban vs rural), and adult equivalent based on estimations derived from the Mexican population.

# **S17 Table- Own- and cross-price elasticity of demand for meat and other food groups comparing lowest to highest income quintile**

*Significant at the 1%. Non-compensated price elasticity standard errors in parentheses.

The ‘Other’ food group category was omitted to avoid collinearity in OLS estimation.

Demand shifters included educational attainment of the head of household, year of survey (2018, 2020, and 2022), region of residence (urban vs rural), adult equivalent based on estimations derived from the Mexican population.

# **S18 Table– Single-day consumption (grams/day) of red and processed meat in ENSANUT 2016 for adult population (≥18 years), by consumption status, and by income quintile**

Values are mean (SE) consumed per day per capita and are weighted to account for complex survey design. Frequencies are weighted to account for complex survey design.

*Animal-source protein foods is the sum of red meat, poultry, eggs, and fish consumption as per recommended 84/day for EAT-Lancet and 140g/day for 2023 Mexican Dietary Guidelines.

** Red meat consumers are those that reported non-zero single-day red meat intake in the 24-hr dietary recall. Processed meat consumers are those that reported non-zero single-day red meat intake in the 24-hr dietary recall.

## **S19 Table - Correlations between changes in Red, Processed, and Total Meat consumption with Non-Meat Food Groups for Period 1 (2006 -2008) and Period 2 (2008 – 2014) in 1,417 Mexican Women**

Bolded p-values are significant at the alpha = 0.05 significance level.
Generalized linear models estimated the change in consumed grams/day between periods for red, processed, and total meat by changes in food group (g/day) consumed for the same period adjusting for changes in total daily energy intake.
