# Supplementary material for: Taxes to red and processed meat to promote sustainable and healthy diets in Mexico
Source: PLoS One. 2025 Jun 27;20(6):e0326616. doi: 10.1371/journal.pone.0326616 (PMC12204545; doi:10.1371/journal.pone.0326616)
Supplement: Attachment — Submitted filename: Response to Reviewers_Connors2025.docx [file pone.0326616.s006.docx]

**Manuscript Title: Promoting adherence to the sustainable Mexican Dietary Guidelines through a tax on red and processed meat**

*We greatly appreciate the careful and thorough consideration of our manuscript. We have incorporated changes as per reviewer comments, which we believe have strengthened the manuscript. Please find our responses to each point below. Note that line numbers refer to those in the manuscript version without tracked changes.*

**Reviewer #1**

First, I would like to commend the authors on a well-conducted and thorough analysis of the effects of price elasticity on the demand for red and processed meat in Mexico. The manuscript provides a detailed exploration of the interactions between price changes and consumer behavior, which is highly relevant for informing public health policies aimed at reducing meat consumption. The consideration of different price increase scenarios effectively highlights the complexity of influencing dietary behaviors through economic interventions, and modeling these effects on a national scale is an important contribution to the literature on food policy and public health.

*Thank you for your careful review of our manuscript, and for highlighting the strengths of this analysis. Below we have addressed suggested changes in-line.*

The manuscript is technically sound in many aspects, with a well-structured methodology and robust statistical analysis. However, there are areas that could benefit from further refinement.

1. Specifically, the interpretation of demand elasticity changes over time would be enhanced by a more in-depth discussion of the socio-economic factors that might influence these trends.

*Response: We agree with the reviewer that there should be we expanded discussion on how socioeconomic factors could explain changes in price elasticities over time [2018, 2020, 2022]. Please find this included in Lines 365 - 372:*

*‘We found that red and processed meat became more inelastic over time (especially after 2020). Shifts in household purchasing patterns due to associated reductions in employment and income following the COVID-19 pandemic could have played a role. An analysis evaluating changes in household purchases from 2018 to 2020 in ENIGH found an increase in basic food purchases: specifically, an increase in processed meat purchases (by approximately 18%) in the lowest income quintile and an increase in SSB purchases (grouped with discretionary foods in our analysis and a complement to red and processed meat) in the rural population in Mexico (33). Although, the proportion of household income allocated to food did not change between these two time periods.’*

*Relatedly, we have included additional detail on socioeconomic factors that may influence the relationship between price and demand of food groups. As one approach, we accounted for this in our modeling of the Mexican food demand system by adjusting for demand shifters – variables that may influence the relationship between price and demand – in our estimation of uncompensated price elasticities of demand.*

*The the motivation for accounting for demand shifters in the demand system is now more clearly explained in Lines 105- 109:*

*‘We accounted for potential sociodemographic factors that could influence the relationship between price and demand through accounting for demand shifters in the model. These included educational attainment of the head of household, year of survey (2018, 2020, and 2022), region of residence (urban vs rural), and adult equivalent based on estimations derived from the Mexican population considering age and size composition of the household (23).’*

*We also stratified by socioeconomic and consumer characteristics to explore whether these characteristics would modify the relationship between price and demand for the food groups included in our analysis. We have stated this more clearly in Lines 109-113:*

*‘Price elasticities were first presented by pooling three survey rounds. We then calculated price elasticities stratified by: (1) survey round, (2) income quintile, (3) consumers of red meat (non-zero expenditures on red meat), and (4) consumers of processed meat (non-zero expenditures on processed meat) to estimate potentially heterogenous impacts on purchases associated with price increases on different consumer groups.’*

2. While the conclusions are generally well-supported by the data, the practical feasibility of the substantial price increases proposed—especially to achieve dietary targets—could be discussed further to address potential consumer responses and implementation challenges.

*Response: Thank you for this comment. Please see below more nuanced discussion on the feasibility, implementation, and potential consumer responses now included in Lines 387-413:*

*‘Our study taken together with the cumulative evidence suggest that large price increases are needed to achieve sufficient reductions in meat consumption, under a critical timeline if we want to meet urgent climate goals such as < 2°C degrees of warming (1) while addressing diet-related health burdens (38). As noted, imposing a tax only on processed meat may be more politically feasible given its stronger association with chronic disease, particularly colorectal cancer and diabetes (2,3,25,28), and that moderate consumption of red meat may still benefit lower income, indigenous populations as a source of nutrients, such as heme iron and vitamin B12 (8). To date, we are only aware of a beverage-specific tax that has been implemented at rates similar to those proposed for processed meat in this study, namely in Saudia Arabia and the United Arab Emirates (39). The food tax in Colombia is expected to increase to 20% by 2025 making it one of the highest food tax rates, globally (36). Factors such as public acceptance, feasibility, and implementation are important. Ensuring that dietary substitutes are available and affordable are critical for feasibility. Furthermore, several conditions may favor such a policy. Mexico currently has political momentum to pass progressive food legislation in favor of health and sustainability. Beyond incorporating sustainability into the national dietary guidelines as a policy tool to justify enforcement, Mexico implemented front-of-package labeling with nutrient warnings on packaged foods and beverages in 2020 (40), and has recently passed a ban on the sale of ultraprocessed foods and beverages in schools to be implemented in March 2025 (41). Additionally, Mexico enacted a General Law on Adequate and Sustainable Nutrition in 2024 that constitutionally protects the right to adequate, nutritious, and sustainably produced food (42), which may serve as a normative framework for complementary legislation in the future. The high suggested price increases in this study are warranted considering the concurrent environmental and health crises, as well as the Mexican government’s present political willingness to act on these issues. These findings serve as an ambitious scenario to understanding the tax rates needed to achieve sufficient changes in consumption across the Mexican population to meet health and environmental objectives. Ambitious targets may serve as a bargaining tool or escalate urgency, given that in practice, negotiation with government, industry, and the public often result in tax rates or policy measures that can fall short of what is deemed necessary by evidence (43–45).’*

*As well as in lines 424- 436:*

*‘On average, adherence to the 2023 Mexican Dietary Guidelines is less expensive than the current diet (by 21%) in the general population since costs are offset by red meat consumption. However, the 2023 Mexican Dietary Guidelines diet was more expensive than the current diet among lower income groups mostly due to an increase in expenditure on nuts, fruits, and vegetables (12). Therefore, special consideration should be taken to ensure the affordability of sustainable and healthy diets, including dietary substitutes, in this population. Again, taxing only processed meat does not pose a risk to diet quality, as its associated health risks outweigh potential benefits. Therefore, this approach may also help mitigate concerns regarding affordability of a ‘healthy’ diet among lower income populations. Nutrition surveillance of at-risk populations is critical to inform how compensatory measures may be designed to enhance dietary quality. Tax revenue recycling in the form of targeted lump sum transfers can be used to benefit lower income groups and counteract any potential regressivity of the tax (47), subject to Congress and government approval as, in Mexico, revenues are not earmarked.’*

*And lines 475- 489:*

*‘Investigation into social determinants of red and processed meat consumption could enhance the effectiveness of food tax policies. For instance, knowledge of within-country cultural differences could be leveraged to tailor targeted policies. Red meat consumption, for example, is higher in Northern and urban regions of the country (12) where it is more prominently featured in staple dishes and tied to culinary identity (52). However, there is reframing of this narrative in efforts seeking to revive the traditional Mexican diet – such as the 2023 Mexican Dietary Guidelines – as the popularity of traditional foods like beans has declined, while meat consumption has increased, particularly after the enactment of the North American Free Trade Agreement in 1994 (53,54). Expanding on these complementary public relations campaigns – such as those led by the Center for Research on Nutrition and Health in Mexico – could highlight food substitutes that are culturally tailored to regional dishes as well as the nutritional and environmental implications to encourage these dietary swaps. Moreso, research on whether lower income families will purchase and cook dietary substitutes is essential in addition to better understanding the nutritional implications of such reductions, ensuring that substitution patterns adequately deliver energy and nutrient requirements.’*

3. The manuscript largely adheres to reporting standards, providing transparency in data sources and methodologies. However, while the statistical significance of the findings is frequently noted, there is insufficient discussion of their practical implications. Expanding on how these statistically significant results might translate into real-world outcomes would greatly enhance the reader's understanding of their relevance and potential impact.

*Response: Thank you for raising this. This complements the response to your second point, which we have pasted a response for above.*

*We have also expanded on practical implications in discussing how this tax may have differential implications across socioeconomic groups [Lines 416-445].*

*‘Given that lower income groups allocate a smaller proportion of their food budget to meat – despite being more price sensitive – the tax would not necessarily impose a disproportionate burden on them. Moreover, lower income groups tend to have greater adherence to the sustainable and healthy traditional Mexican Diet, which serves as a foundation for Mexican National Dietary Guidelines (8,12). Therefore, red and processed meat consumption in this group would change by a smaller proportion, and food substitutions would align more closely with traditional dietary patterns as was observed in our models (12). In the case of health-motivated taxes, reductions in consumption among lower income groups can actually mitigate health disparities and healthcare expenditures (46). On average, adherence to the 2023 Mexican Dietary Guidelines is less expensive than the current diet (by 21%) in the general population since costs are offset by red meat consumption. However, the 2023 Mexican Dietary Guidelines diet was more expensive than the current diet among lower income groups mostly due to an increase in expenditure on nuts, fruits, and vegetables (12). Therefore, special consideration should be taken to ensure the affordability of sustainable and healthy diets, including dietary substitutes, in this population. Again, taxing only processed meat does not pose a risk to diet quality, as its associated health risks outweigh potential benefits. Therefore, this approach may also help mitigate concerns regarding affordability of a ‘healthy’ diet among lower income populations. Nutrition surveillance of at-risk populations is critical to inform how compensatory measures may be designed to enhance dietary quality. Tax revenue recycling in the form of targeted lump sum transfers can be used to benefit lower income groups and counteract any potential regressivity of the tax (47), subject to Congress and government approval as, in Mexico, revenues are not earmarked. They can be used to subsidize the cost of healthier, more nutrient-rich foods such as F&V and legumes especially since both food groups were stronger substitutes to red and processed meat in the lowest compared to highest income group. This was explored in a recent study evaluating the regressivity of meat taxes across Europe (47). It found that tax recycling through lump sum transfers to the lowest quintile were more equitable than lowering the price of fruits and vegetables (47). Moreover, a social-cost benefit analysis applied price elasticities obtained from a literature review to test price increases of 15 and 30% to total meat (including red, white and processed) and found that a 10% decrease in price of fruit and vegetables counteracted regressivity among lower income groups (17). In addition to financial incentives, nutritional education may be a complementary strategy to ensure that these populations are making the ‘healthier’ choice.’*

*And an additional caveat in Lines 345-346:*

*‘Therefore, increasing the price of red and processed meat may promote greater adherence to the guidelines, especially in lower income groups, which can improve diet quality.’*

**Reviewer #2**

*Response: We greatly appreciate the time the reviewer spent in evaluating the manuscript and providing the valuable suggestions below, which have contributed to the strengthening the impact of this analysis.*

1. The manuscript needs further clarification and enhancement in some key areas. For example, the methodology, especially the estimation of price elasticities, would be enhanced by a detailed discussion of assumptions made during the analysis. This might include suggestions for investigating possible confounding variables-what affects consumer behavior other than price changes, such as cultural considerations or alternative sources for food products. This could also involve sensitivity analyses that prove the strength of the results found.

*Thank you, in terms of technical assumptions made in the modeling, we have now provided more detail in Lines 99- 105:*

*‘The linear approximate model was chosen to model a linear relationship between price and quantity demanded for interpretability and consistency with prior food literature (20–22). The model was estimated using Ordinary Least Squares (OLS) equation and ‘other foods’ was omitted to account for potential collinearity between food groups. Estimation using OLS assumes that errors are normally distributed and that there is no correlation in errors between equations (20). We also restricted the model to comply with the homogeneity and symmetry constraints, and verified that the adding-up property was met (20).’*

*We have also conducted a sensitivity analysis estimating the linear approximate of the almost demand system with Seemingly Unrelated Regression instead of Ordinary Least Squares to validate the robustness of the estimated price elasticities [Lines 131 -135; S18 Table]*

*‘In the second, we estimated the demand system using the Seemingly Unrelated Regression (26) – which accounts for correlation in the error terms across food group equations – to compare with results estimated from OLS, that omitted the ‘other foods’ group, to ensure that our estimations of the price elasticities of demand were robust.’*

*Results of sensitivity analysis in Lines 226-234:*

*‘Price elasticities estimated with Seemingly Unrelated Regression*

*The own-price elasticities estimated using Seemingly Unrelated Regression in the sensitivity analysis were broadly consistent for red and processed meat, although slightly more inelastic than those found in our primary analysis (-0.61 and -0.75, respectively; Table S18). Own-price elasticities were similar for the remaining the food groups modeled in the system. Cross-price elasticities were mostly consistent between both estimation methods; however, slight differences were observed (Table S18). In the sensitivity analysis, dairy and legumes were complements to red meat whereas they were weak substitutes in the primary analysis. Moreover, for processed meat, no relationship was found between the price of processed meat and demand for discretionary compared to a weak complement in the primary analysis.’*

*Additionally, beyond the demand shifters that were accounted for in our demand system, we have now included discussion of other potential determinants of consumption including culture and availability/acceptability of substitutes that may affect consumer preferences in Lines 472-493:*

*‘Future studies should examine the potential to explore combined policy mechanisms such as front-of-package labeling or signaling (i.e., public framing and messaging) in combination with taxes given combined policy mechanisms have been found to be more effective in trial settings since price is not the only determinant of food choice (16,50,51). Investigation into social determinants of red and processed meat consumption could enhance the effectiveness of food tax policies. For instance, knowledge of within-country cultural differences could be leveraged to tailor targeted policies. Red meat consumption, for example, is higher in Northern and urban regions of the country (12) where it is more prominently featured in staple dishes and tied to culinary identity (52). However, there is reframing of this narrative in efforts seeking to revive the traditional Mexican diet – such as the 2023 Mexican Dietary Guidelines – as the popularity of traditional foods like beans has declined, while meat consumption has increased, particularly after the enactment of the North American Free Trade Agreement in 1994 (53,54). Expanding on these complementary public relations campaigns – such as those led by the Center for Research on Nutrition and Health in Mexico – could highlight food substitutes that are culturally tailored to regional dishes as well as the nutritional and environmental implications to encourage these dietary swaps. Moreso, research on whether lower income families will purchase and cook dietary substitutes is essential in addition to better understanding the nutritional implications of such reductions, ensuring that substitution patterns adequately deliver energy and nutrient requirements. Additionally, a political analysis examining the key stakeholders and political instruments that would facilitate the passing of such legislation – as was done for the 2014 tax in Mexico (44) – would be beneficial considering the large reductions in demand required to meet sustainable and healthy dietary targets. Lastly, research should estimate the impact of these taxes on environmental outcomes.’*

2. Secondly, there should be more discussion on implications related to the most vulnerable populations and increases in meat prices. While it is mentioned that consumers of meat from a low-income background are more responsive to any changes in price, some indication should be made concerning how this population can be supported in making healthier choices, given rising prices. This needs to be in regard to certain policy measures or even subsidies in terms of healthier alternatives. This, in turn, would allow a more detailed exploration of how meat taxation could impact both public health and equity.

*Response: Agreed. We have further emphasized that, given the evidence, raising the price of processed meat may be the the most equitable option since it raises no concerns regarding impacting the dietary quality of lowest income groups. Second, we have also added additional emphasis regarding the need for affordable and available healthy dietary substitutes and policy measures that may undertaken to support this. We have expanded in Lines 416-445 to include:*

*‘Given that lower income groups allocate a smaller proportion of their food budget to meat – despite being more price sensitive – the tax would not necessarily impose a disproportionate burden on them. Moreover, lower income groups tend to have greater adherence to the sustainable and healthy traditional Mexican Diet, which serves as a foundation for Mexican National Dietary Guidelines (8,12). Therefore, red and processed meat consumption in this group would change by a smaller proportion, and food substitutions would align more closely with traditional dietary patterns as was observed in our models (12). In the case of health-motivated taxes, reductions in consumption among lower income groups can actually mitigate health disparities and healthcare expenditures (46). On average, adherence to the 2023 Mexican Dietary Guidelines is less expensive than the current diet (by 21%) in the general population since costs are offset by red meat consumption. However, the 2023 Mexican Dietary Guidelines diet was more expensive than the current diet among lower income groups mostly due to an increase in expenditure on nuts, fruits, and vegetables (12). Therefore, special consideration should be taken to ensure the affordability of sustainable and healthy diets, including dietary substitutes, in this population. Again, taxing only processed meat does not pose a risk to diet quality, as its associated health risks outweigh potential benefits. Therefore, this approach may also help mitigate concerns regarding affordability of a ‘healthy’ diet among lower income populations. Nutrition surveillance of at-risk populations is critical to inform how compensatory measures may be designed to enhance dietary quality. Tax revenue recycling in the form of targeted lump sum transfers can be used to benefit lower income groups and counteract any potential regressivity of the tax (47), subject to Congress and government approval as, in Mexico, revenues are not earmarked. They can be used to subsidize the cost of healthier, more nutrient-rich foods such as F&V and legumes especially since both food groups were stronger substitutes to red and processed meat in the lowest compared to highest income group. This was explored in a recent study evaluating the regressivity of meat taxes across Europe (47). It found that tax recycling through lump sum transfers to the lowest quintile were more equitable than lowering the price of fruits and vegetables (47). Moreover, a social-cost benefit analysis applied price elasticities obtained from a literature review to test price increases of 15 and 30% to total meat (including red, white and processed) and found that a 10% decrease in price of fruit and vegetables counteracted regressivity among lower income groups (17). In addition to financial incentives, nutritional education may be a complementary strategy to ensure that these populations are making the ‘healthier’ choice.’*

*And in Lines 444-445:*

*‘In addition to financial incentives, nutritional education may be a complementary strategy to ensure that these populations are making the ‘healthier’ choice.’*

*Lines 486-489:*

*‘Moreso, research on whether lower income families will purchase and cook dietary substitutes is essential in addition to better understanding the nutritional implications of such reductions, ensuring that substitution patterns adequately deliver energy and nutrient requirements.’*

*Lines 496-498:*

*‘Specifically, substantially increasing the price of processed meat may be the best option for maximum health gains, and little risk to diet quality, while reducing environmental costs.’*

**Reviewer #3**

First of all, I would like to express my thankful for the editor of EJVS for their trust in reviewing this manuscript. This article is very interesting in the field of food safety in Mexico.

The introduction covers the problem, statement, and objectives of the study.
Material and methods explain in detail how authors collected and analyzed their data.
Results and discussion section are clearly and reflect the most abundant findings
Note: change all table and figure titles to understand more, change figure resolution.

*Response: We greatly appreciate your enthusiasm for this work. Following your suggestion, we have corrected the figure/table titles and resolution of the images to be more interpretable.*

**Reviewer #4**

This is an economic report not a scientific paper
We assessed how to achieve sustainable, equitable, and healthy dietary targets for red and processed meat consumption in Mexico. The four specific aims were to (1) estimate own- and cross-price elasticities of red and processed meat, (2) estimate demand responses to price increases in red and processed meat under various scenarios and apply them to consumption data, (3) explore if price elasticities varied by household income and other effect modifiers (i.e., survey year, and consumption status) and (4) corroborate cross-sectional findings by evaluating dietary substitutions and complements to red and processed meat in a longitudinal cohort of Mexican women.
REJECT

*Response: Your time in reviewing our article is appreciated. We note that other than the first sentence the reviewer’s comment reproduces the text from the fourth paragraph of the manuscript’s Introduction.*

*We respectfully disagree that this is not a scientific paper. Below we outline the scientific contributions of this work, highlighting how the article relates to the scientific literature.*

1. ***Estimation of Mexican Food Demand System****: This analysis seeks to inform the design of fiscal mechanisms aimed at improving public health and environmental outcomes. The calculation of price elasticities of demand is a powerful scientific research tool that can be used to guide the design and evaluate specific food policies, especially taxes, as demonstrated previously (Colchero et al 2015; Guerrero-López et al 2017; Caro et al 2017). Another contribution of this research is that we modeled red and processed meat alongside other key food groups within Mexican food system. This allowed for greater understanding of potential impacts to the rest of the diet, that could impact nutrition and the environment, which is unique meat tax literature.*
2. ***Estimating changes in consumption according to proposed policies:*** *Comparing current population consumption with policy benchmarks, such as Dietary Guidelines, is useful in that it allows scientists and policymakers to evaluate the implementation gap as well as study variations in policy-reach across segments of the population (Castellanos-Gutiérrez et al 2021; Unar-Munguía et al 2024). A key scientific contribution of this work is to address this implementation gap through evaluating fiscal policy mechanisms and integrating epidemiological data to support this evidence. This approach demonstrates how policy can promote dietary changes that aligns population-level consumption with policy guidelines, thereby estimating real-word impacts. Moreover, understanding post-policy consumption lays a foundation of evidence that can be used to predict potential health and environmental impacts in future work, as has been done in prior research (Broeks et al 2020; Springmann et al 2018; Springmann et al 2025; Schönbach et al 2019).*
3. ***Beyond economic modeling, evaluation of dietary patterns over time****: We additionally carried out an exploratory analysis of potential dietary substitution and complement behaviors over time using dietary intake data in a longitudinal Mexican cohort. This strengthens the scientific validity of our work as we were able to corroborate findings from the economic model with observational dietary behaviors.*

*In sum, we are confident that our paper makes a strong, and timely scientific contribution to the academic literature.*

*Broeks MJ, Biesbroek S, Over EAB, van Gils PF, Toxopeus I, Beukers MH, et al. A social cost-benefit analysis of meat taxation and a fruit and vegetables subsidy for a healthy and sustainable food consumption in the Netherlands. BMC Public Health. 2020 May 11;20(1):643.*

*Caro, Juan Carlos, et al. "Designing a food tax to impact food-related non-communicable diseases: the case of Chile." Food Policy 71 (2017): 86.*

*Castellanos-Gutiérrez A, Sánchez-Pimienta TG, Batis C, Willett W, Rivera JA. Toward a healthy and sustainable diet in Mexico: where are we and how can we move forward? Am J Clin Nutr. 2021 May 1;113(5):1177–84.*

*Colchero, M. Arantxa, et al. "Price elasticity of the demand for sugar sweetened beverages and soft drinks in Mexico." Economics & Human Biology 19 (2015): 129-137.*

*Guerrero-López, Carlos M., Mishel Unar-Munguía, and M. Arantxa Colchero. "Price elasticity of the demand for soft drinks, other sugar-sweetened beverages and energy dense food in Chile." BMC public health 17 (2017): 1-8.*

*Unar-Munguía M, Cervantes-Armenta MA, Rodríguez-Ramírez S, Bonvecchio Arenas A, Fernández Gaxiola AC, Rivera JA. Mexican national dietary guidelines promote less costly and environmentally sustainable diets. Nat Food. 2024 Aug;5(8):703–13.*

*Schönbach, Johanna-Katharina, Silke Thiele, and Stefan K. Lhachimi. "What are the potential preventive population-health effects of a tax on processed meat? A quantitative health impact assessment for Germany." Preventive Medicine 118 (2019): 325-331.*

*Springmann M, Mason-D’Croz D, Robinson S, Wiebe K, Godfray HCJ, Rayner M, et al. Health-motivated taxes on red and processed meat: A modelling study on optimal tax levels and associated health impacts. PloS One. 2018;13(11):e0204139.*

*Springmann, Marco, et al. "A reform of value-added taxes on foods can have health, environmental and economic benefits in Europe." Nature Food (2025): 1-9.*
