# Supplementary material for: Taxes to red and processed meat to promote sustainable and healthy diets in Mexico
Source: PLoS One. 2025 Jun 27;20(6):e0326616. doi: 10.1371/journal.pone.0326616 (PMC12204545; doi:10.1371/journal.pone.0326616)
Supplement: Attachment — Submitted filename: Dear Editor.docx [file pone.0326616.s007.docx]

Dear Editor

**Manuscript title: Promoting adherence to the sustainable Mexican Dietary Guidelines through a tax on red and processed meat**

My comment on the manuscript review was “This is an economic report, not a scientific paper” and this paragraph *“We assessed how to achieve sustainable, equitable, and healthy dietary targets for red and processed meat consumption in Mexico. The four specific aims were to (1) estimate own- and cross-price elasticities of red and processed meat, (2) estimate demand responses to price increases in red and processed meat under various scenarios and apply them to consumption data, (3) explore if price elasticities varied by household income and other effect modifiers (i.e., survey year, and consumption status) and (4) corroborate cross-sectional findings by evaluating dietary substitutions and complements to red and processed meat in a longitudinal cohort of Mexican women”* was copied from the manuscript to clear the objective of the present paper according to the opinion of the authors.

I appreciate these goals, but this study used only publicly available secondary data and did not include experimental work regarding Animal Physiology or meat technology.

This work presents academic information. Therefore, my opinion may not be correct according to my scientific trend. So, I am sorry to re-review this manuscript.

Regards

Abdel-Khalek E. Abdel-Khalek
