# Supplementary material for: Taxes to red and processed meat to promote sustainable and healthy diets in Mexico
Source: PLoS One. 2025 Jun 27;20(6):e0326616. doi: 10.1371/journal.pone.0326616 (PMC12204545; doi:10.1371/journal.pone.0326616)
Supplement: Attachment — Submitted filename: Response_to_Reviewers_Connors2025_auresp_2.docx [file pone.0326616.s008.docx]

**Manuscript Title: Promoting adherence to the sustainable Mexican Dietary Guidelines through a tax on red and processed meat**

*Thank you for providing further comments to the manuscript. We have kindly addressed them below. Please note that the line numbers refer to the manuscript version with untracked changes.*

**Reviewer #4**

Dear authhors

Manuscript title: Promoting adherence to the sustainable Mexican Dietary Guidelines through a tax on red and processed meat
My comment on the manuscript review was “This is an economic report, not a scientific paper” and this paragraph “We assessed how to achieve sustainable, equitable, and healthy dietary targets for red and processed meat consumption in Mexico. The four specific aims were to (1) estimate own- and cross-price elasticities of red and processed meat, (2) estimate demand responses to price increases in red and processed meat under various scenarios and apply them to consumption data, (3) explore if price elasticities varied by household income and other effect modifiers (i.e., survey year, and consumption status) and (4) corroborate cross-sectional findings by evaluating dietary substitutions and complements to red and processed meat in a longitudinal cohort of Mexican women” was copied from the manuscript to clear the objective of the present paper according to the opinion of the authors.
I appreciate these goals, but this study used only publicly available secondary data and did not include experimental work regarding Animal Physiology or meat technology.
This work presents academic information. Therefore, my opinion may not be correct according to my scientific trend. So, I am sorry to re-review this manuscript.

*We thank the reviewer for clarifying their original comments and are sorry that our scientific discipline’s approach differs from the expectations of the reviewer. This is an epidemiological study using nationally representative data and well-* *established, rigorous economics modeling. “Animal Physiology” and “meat technology” are not appropriate disciplines to answer the above-stated objectives on price elasticities, demand responses, and human consumption behavior.*

**Reviewer #5**

This study makes a valuable contribution to the literature on food taxes and sustainable diets, particularly in the context of middle-income countries like Mexico. Its strengths lie in its robust methodology, focus on equity, and actionable policy recommendations. However, I would recommend minor revisions to strengthen the study before publication.

While the study highlights substitution patterns, it does not fully explore the potential nutrient gaps that might arise from reduced meat consumption, especially for vulnerable groups (e.g., iron and vitamin B12 deficiencies). A more detailed nutrient analysis would strengthen the study.

*We greatly appreciate this input and agree that potential nutrient gaps are an important consideration. This was the motivation for our investigation into potential dietary substitutes and complements, given a diverse diet is an important predictor of micronutrient adequacy. For this reason, evaluating the entire diet is key. Recognizing this, the Mexican Dietary Guidelines emphasizes a diverse diet, replacing red and processed meat with beans, lentils, egg, poultry, and fish which we have evaluated here. Moreover, the guidelines have been designed to provide a micronutrient adequate diet. We have further elaborated on this in lines 345-348:*

*‘Therefore, increasing the price of red and processed meat may promote greater adherence to the guidelines, especially in lower income groups, which has been designed to deliver a micronutrient adequate diet, and therefore, may improve diet quality due to increases in consumption of nutrient-dense foods and reductions in discretionary foods.’*

*Additionally motivated by this point, we provided estimations regarding potential changes to consumption of food groups rich in iron and vitamin B12 – such as animal-sourced protein foods – to incorporate flexibility in real-life dietary substitutions. We found that consumption was predicted to increase following price increases to red and processed meat across all four scenarios due to substitution behaviors (Table 2).*

*However, given the heterogeneity of foods within these broad food groups and that we did not model changes in consumption of the rest of the diet, determining the specific changes in nutrient intakes is out of the scope of this research and has been emphasized as a direction for future research in lines 493-501:*

*‘Moreso, research on whether lower income families will purchase and cook dietary substitutes is essential in addition to better understanding the nutritional implications of such reductions, ensuring that substitution patterns adequately deliver energy and nutrient requirements. Moreover, further research into the nutrient implications resulting from reductions in red and processed meat – especially for iron and vitamin B12 – while considering substitutions in the rest of the diet is a key priority, especially among lower income populations. Adherence to the Mexican Dietary Guidelines and a diverse diet is indicative of micronutrient adequacy, not just red and processed meat dietary targets, therefore, this should be considered in future micronutrient evaluations.’*

*We have also added additional consideration on policies that be designed to promote the consumption of nutrient-rich foods (particularly those rich in vitamin B12 and iron) that target groups most at-risk of nutrient inadequacy such as food vouchers. Please see lines 438-441:*

*‘They can be used to subsidize the cost via subsidies, food vouchers, or cash transfers to enable purchasing of healthier, more nutrient-rich foods such as F&V and legumes, especially since both food groups were stronger substitutes to red and processed meat in the lowest compared to highest income group.’*

*And lines 447-452:*

*‘Food vouchers may facilitate more direct targeting of subgroups most at-risk of nutrient inadequacy and be designed for the purchase of nutrient-rich foods, particularly rich in iron and vitamin B12, such as seafood, poultry, or eggs. In a randomized study conducted in Ecuador, food vouchers were found to increase dietary diversity as opposed to in-kind food or cash assistance[48]. In addition to financial incentives, nutritional education may be a complementary strategy to ensure that these populations are making the ‘healthier’ choice.’*

*Our final point is that we also found that lower income groups would have less ‘drastic’ changes to their diet since they currently purchase less red and processed meat compared to higher income groups in Mexico as discussed in lines 418-424:*

*‘Given that lower income groups allocate a smaller proportion of their food budget to meat – despite being more price sensitive – the tax would not necessarily impose a disproportionate burden on them. Moreover, lower income groups tend to have greater adherence to the sustainable and healthy traditional Mexican Diet, which serves as a foundation for Mexican National Dietary Guidelines [8,12]. Therefore, red and processed meat consumption in this group would change by a smaller proportion, and food substitutions would align more closely with traditional dietary patterns as was observed in our models [12].’*
