# Supplementary material for: Taxes to red and processed meat to promote sustainable and healthy diets in Mexico
Source: PLoS One. 2025 Jun 27;20(6):e0326616. doi: 10.1371/journal.pone.0326616 (PMC12204545; doi:10.1371/journal.pone.0326616)
Supplement: Attachment — Submitted filename: Response to Editor_Connors2025.docx [file pone.0326616.s009.docx]

**Proposed Revised Title: Taxes to red and processed meat to promote sustainable and healthy diets in Mexico**

*We greatly appreciate the Editor’s thorough review of work and comments that have elevated the quality of the work. We have responded to comments line-by-line below. Line numbers refer to the version with untracked changes.*

**Additional Editor Comments:**

The authors evaluated how price increases to red and processed meat could shift consumption for meat as well as other key food groups, and did this by using data from the Mexican National Household Income and Expenditure Survey (2018, 2020, 2022). The revisions done thus far has elevated the quality of the work. However, the editor suggests further revisions are needed before it can be accepted.

a) After several reads, I deeply worry that the title does not strongly reflect the objective of the work as stated above. I suggest title: Price increases of red and processed meat - Would it shift consumption to other foods?
Authors, think deeply about this, I believe the simpler the title, the better it is to your work. Also, this title will attract readers around the globe, plus citations.

*Thank you for the title suggestion. The primary aim of our study was to explore how policies – specifically fiscal policies – could be used to achieve sustainable and healthy dietary targets in the Mexican population. While we were interested in shifts in consumption of other food groups to give us an understanding of potential changes to the entire diet, our primary focus was not whether these would shift (which was expected in the demand system) but how to promote adherence to specific dietary targets.*

*To better reflect this focus as well as expand readership, we propose the following revised title:*

*‘Taxes to red and processed meat to promote sustainable and healthy diets in Mexico’*

b) The introduction needs to be totally revised. Many aspects of current version needs to be removed. Kindly follow this path, to build three strong paragraphs.

- Paragraph one should introduce price elasticity applied to food produce, what is the global debate, are they agreements, and disagreements, which food produce are more debated, make sure to mention those of animal food products.
- Paragraph two should then narrow down to price elasticity arguments as it relates to meat products, what are the sustainability implications, (you can mention emissions, healthy diet, government policy but it must be on a global perspective, and not narrowed to any country specifically.
- Paragraph 3 should then introduce Mexico, population, meat production, and existing policies that have helped accumulate government data. Then, create the hypothesis and gap in existing knowledge (paucity of published information regards how price increases to red and processed meat could shift consumption for meat as well as other key food groups. This is a more robust approach to state why estimates own- and cross-price elasticities could help provide better understanding of the accumulated government data.

*Thank you for the suggested revisions to the introduction. We have restructured the introduction as per request while maintaining emphasis on the objective of this study and food taxes rather than the methods employed to address the research question. Please find the revised introduction in Lines 53-94:*

*‘Government-supported demand-side interventions, such as fiscal policies, are one way to support food system transformations and behavior change [1]. Food-specific taxes have been most commonly applied to sugar-sweetened beverages (SSBs), nonessential energy-dense foods, and saturated fat to address diet-related chronic disease burdens [2–4]. Taxes have indeed been effective in reducing targeted food purchases [4,5], therefore, may serve as a policy tool to achieve double-duty goals for health and sustainability. Estimating price elasticities enhances empirical understanding of demand responses to price increases of taxed commodities as well as potential substitutes and complements to those commodities, and thus have been calculated to inform tax policy design [6,7].*

*Meat production accounts for a majority of food system emissions, accounting for one third of greenhouse gas emissions [8]. Simultaneously, high consumption of red and processed meat is also associated with colorectal cancer [9] and type 2 diabetes [10], among other health risks [11,12]. Moderating levels of meat consumption is central to sustainable and healthy diets [13,14], particularly in high- and middle- income contexts where meat consumption is disproportionately high [15,16]. Several studies have estimated or utilized price elasticities to model hypothetical taxes on meat in Europe, the United States, and more recently, Brazil for precisely this purpose [17–21]. Demand responses to price vary by population and commodity; curbing demand for red and processed meat has been found to require more aggressive approaches such as higher rates of taxation as well as varying rates across consumer groups and geographies [17,18]. Thus, derivation of price elasticities to estimate demand responses to such policies is a practical tool that may inform the magnitude of increases that would be needed to achieve desired consumption levels.*

*Global diets require drastic transformations to keep within net zero emissions targets and alleviate significant diet-related disease burdens [8,22]. Recognizing this, Mexico integrated sustainability into the national Dietary Guidelines [14]. Nevertheless, adherence to sustainable and healthy diets remains low in Mexico [23,24] despite research suggesting that such diets are affordable in this context [25,26]. In particular, meat consumption continues to increase and is well above recommended levels [23]. Thus, reducing red and processed meat consumption has been identified as key to increasing the sustainability of healthy diets in the Mexican context [23,24]. Mexico was one of the first countries to pass a food-specific tax in 2014 on SSBs and an ad valorem tax on non-essential energy-dense foods motivated by increasing diet-related chronic disease burdens such as obesity and type 2 diabetes [5]. The path toward a sustainable and healthy diet in Mexico has been clearly outlined [14] and assessed for feasibility and affordability [26], but an implementation gap remains that necessitates government policy intervention. Building on Mexico’s food policy toolkit [27], a tax to red and processed meat can help address this gap. To date, such a tax has not been studied in the Mexican context and understanding of how it might shift consumption in the rest of the diet -- essential to diet quality -- is not well understood.*

*We assessed how to achieve sustainable, equitable, and healthy dietary targets for red and processed meat consumption in Mexico. The four specific aims were to (1) estimate own- and cross-price elasticities of red and processed meat, (2) estimate demand responses to price increases in red and processed meat under various scenarios and apply them to consumption data, (3) explore if price elasticities varied by household income and other effect modifiers (i.e., survey year, and consumption status) and (4) corroborate cross-sectional findings by evaluating dietary substitutions and complements to red and processed meat in a longitudinal cohort of Mexican women.’*

c) Please, your materials and methods should start with a new subsection captioned "Schematic overview of evaluation program", which must have a flow diagram that shows the major steps of methods, and at least 4 sentences. Sentence one must introduce the flow diagram. Sentence two presents the major steps, and connects it to the overall aim of the work. Sentence three explains why this method is robust, and make sure you support it with relevant literature. Sentence 4 clarifies the steps taken to ensure validity and authenticity of the work.

*Thank you for this helpful suggestion. We would like to clarify that our study did not evaluate a program but rather modeled the potential impact of a food policy intervention. Thus, referring to a ‘schematic overview of evaluation program’ can be very misleading to readers. Please let us know if you have any comments in follow-up.*

d) I examined the remaining aspects of the methods. It is very ok. Make sure you end the methods with a new subsection "Statistical analysis" ...provide ample detail for this, show all the formulae for price estimate procedures, provide supporting references, please provide ample detail that is very sufficient, and convincing

*Thank you, we have now included a section titled, ‘Statistical Analyses’, with model specifications and price index estimation in Lines 118-169.*

*‘We calculated uncompensated own- and cross-price elasticities of demand for 10 food groups through estimation of a complete demand system using a linear approximate of an Almost Ideal Demand System (LA/AIDs)[28]. The linear approximate model was chosen to model a linear relationship between price and quantity demanded for interpretability and consistency with prior food literature [28–30]. Briefly, the model calculates a set of estimates based on the proportion of the budget dedicated to a particular food group out of the total household food expenditures. These estimates approximate the response in demand to changes in the price of a food group without considering changes in income.*

*Price was calculated based on the quantity purchased (kg or liters) and the expenditures reported for that quantity of the food group. Prices were then averaged at the municipality level to reduce potential record biases at the household level [6]. Outliers at the municipality level that fell ±2 standard deviations from the national mean were replaced with the weighted average national price for that food group (n=276 [0.4%] for 2018; n=166 [0.2%] for 2020; and n=138 [0.2%] for 2022). The expenditure share of each food group was calculated as the sum of expenditures in each food group divided by total household food expenditure.*

*The LA/AIDs model is defined as:*

(1) $w_{hgmt}$ *=* $\alpha_{g}+$ $\sum_{j=1}^{j} \beta_{gi}\ln p_{mjt}$ *+* $\gamma$ *ln* $\frac{E}{P*}$ *+* $\sum_{v=1}^{v} \delta_{gv} \eta_{hmtv}$ *+* $\mu_{hmgt}$

*LA/AIDs model	Covariate term*

*As specified,* $w_{ghmt}$ *is the proportion of household expenditures spent on a particular food group g for household h residing in municipality m in survey year t. Each individual food item within each food group is denoted as j and* $p_{mjt}$ *represents the price of each food item at the municipality level m in the year t the survey was administered. E represents total household expenditures on food while lnP* is the Laspeyres price index represented as ln*$P_{jt}$ *in Equation 1 above. The covariate term* $\eta$ *denotes shifter variables which may affect demand for a good,* $v,$ *at the household and municipality level that will be added to the model. Lastly,* $\mu$*is the error term for the model.*

*We included the Laspeyres Price Index to account for changes in price over time and maintain linearity in the parameters by weighting price according to baseline prices and shares (in this case, 2018) for a constant basket of foods[31]. The index is calculated as:*

(1) *ln*$P_{jt}$ *=* $\sum_{g=1}^{j-1} \bar{w_{g}}$ ** ln* $p_{mjt}$

$p_{mjt}$ *is defined as the price per unit for each jth food item in municipality m at survey year t. In addition,* $\bar{w_{g}}$ *is defined as the average budget share for the food group g pertaining to food item j. More detailed information on the price elasticity estimation can be found in S1 Appendix.*

*The model was estimated using Ordinary Least Squares (OLS) equation and ‘other foods’ was omitted to account for potential collinearity between food groups. Estimation using OLS assumes that errors are normally distributed and that there is no correlation in errors between equations [28]. We also restricted the model to comply with the homogeneity and symmetry constraints, and verified that the adding-up property was met [28]. We accounted for potential sociodemographic factors that could influence the relationship between price and demand through accounting for demand shifters in the model. These included educational attainment of the head of household, year of survey (2018, 2020, and 2022), region of residence (urban vs rural), and adult equivalent based on estimations derived from the Mexican population considering age and size composition of the household [33]. Adult equivalent has been previously included in food demand system analyses for Mexico [6] to account for how household composition may impact welfare due to household composition and the distribution of income amongst members. Price elasticities were first presented by pooling three survey rounds. We then calculated price elasticities stratified by: (1) survey round, (2) income quintile, (3) consumers of red meat (non-zero expenditures on red meat), and (4) consumers of processed meat (non-zero expenditures on processed meat) to estimate potentially heterogenous impacts on purchases associated with price increases on different consumer groups. More details on model specifications are in S1 Appendix. We used the package for Demand Analysis with the Almost Ideal Demand System in R statistical software to estimate the demand system [34].’*

e) I examined the results, it is very good. There should be more information from Price elasticities of meat by cut quality, even though you did not simulate demand responses to price increases by cut of meat.

*Thank you for this suggestion, we have now included more information on the cross-price elasticities with cuts of meat quality in Lines 272-286.*

‘*The own-price elasticities for cheaper and more expensive cuts of meat were -0.83 and -0.78, respectively (S2 Appendix; S16-S17 Tables; S2 Fig). Eggs and F&V were complements to cheaper cuts of meat whereas they were substitutes for expensive cuts of meat. Seafood and poultry were substitutes for cheaper cuts of meat but complements to expensive cuts. Grains and F&V were stronger substitutes to expensive cuts of meat compared to cheaper cuts and legumes were a stronger substitute to cheaper cuts. Stratified by income quintile, cheaper cuts were more price elastic than expensive cuts in the lowest income group whereas in the top income quintiles the opposite was true (S2 Fig.). Additionally, there were differential cross-price elasticities by income group (S17 Table). Poultry and dairy were stronger substitutes and eggs, discretionary foods, grains, and expensive cuts of meat were stronger complements to cheaper cuts of meat in the highest compared to lowest income group. Whereas, eggs and grains were stronger substitutes, and dairy, discretionary foods and cheaper cuts of meat were stronger complements to expensive cuts of meat in highest compared to lowest income group.*

*Cheaper cuts of meat tended to be processed meat whereas more expensive cuts of meat tended to be red meat (hence, the similarity between own-price elasticities) (S4 Table). Therefore, we did not simulate demand responses to price increases by cut of meat.’*

f) Please, have another look at your discussion, and keep the discussion, strictly discussion. All the tables and figures captured in results, must be referred to in the discussion. Use (Refer to Table ?) or (Refer to Figure ?) where and when a specific data of table or figure is mentioned. I can see you have a lot of supplementary information. I suggest that you develop a figure that tactfully captures key message of various supplementary info, which should be used to support your discussion.

*We are regular readers of PLOS One publications, and have reviewed several recently published articles, and have not seen tables, figures, and supplemental materials explicitly called out in this way in the discussion section. We are happy to do this based on your request, but we wanted to confirm that you wanted us to deviate from what appears to be standard practice to date for PLOS one papers first.*

g) please, remove all aspects of conclusion, and create a section 'Concluding remarks", and move them there. please, remove all aspects of future studies, and move them to a new section 'Recommendations for future work"

*We have now labeled these sections accordingly as suggested, thank you.*

Look forward to your revised manuscript. I believe the quality of your work will be far much better after this revision. Thank you and God bless
